# Efficacy and Safety of Propolis for Treating Recurrent Aphthous Stomatitis (RAS): A Systematic Review and Meta-Analysis

**DOI:** 10.3390/dj12010013

**Published:** 2024-01-06

**Authors:** Tina Roberts, Idriss Ibrahim Kallon, Anel Schoonees

**Affiliations:** 1 Department of Craniofacial Biology, Pathology and Radiology, Faculty of Dentistry, University of the Western Cape, Cape Town 7500, South Africa; 2 Centre for Evidence-Based Health Care, Division of Epidemiology and Biostatistics, Department of Global Health, Faculty of Medicine and Health Sciences, Stellenbosch University, Cape Town 7500, South Africa; kalloni@sun.ac.za (I.I.K.); anelschoonees@sun.ac.za (A.S.)

**Keywords:** efficacy, propolis, safety, recurrent aphthous stomatitis (RAS)

## Abstract

The systematic review assessed the efficacy and safety of propolis for treating recurrent aphthous stomatitis (RAS). The review adopted the PICO framework to examine the effects of topical and systemic propolis on RAS while also comparing it to established treatments, placebos, or no treatment. The main focus was on the healing time, pain levels, adverse effects, the likelihood of ulcer recurrence, and accompanying symptoms such as redness. The team included randomised controlled trials (RCTs) and quasi-randomised trials, excluding case reports and studies on oral ulcers other than RAS. In May 2022, the review team comprehensively searched nine databases and trial registries following the PRISMA guidelines. The protocol was registered in the PROSPERO database under the registration number CRD42022327123. Two review authors conducted a comprehensive and autonomous search for pertinent papers and extracted essential data. Where data permitted, the team utilised Review Manager 5 to conduct a random-effects meta-analysis, assessing the risk of bias and heterogeneity of the included studies. Where possible, the GRADE Pro programme was used to assess the certainty of the evidence for all the outcomes. This review included 10 RCTs, comprising 825 participants aged between 18 and 69 years. Seven studies evaluated the efficacy and safety of propolis when applied topically, all of which used different formulations, concentrations, and carriers. The remaining three studies assessed systemic administration in tablet form. The duration of investigations ranged from 5 days to 3 years. The review team classified two studies as having an overall ‘high risk’ of bias, while the remaining studies were categorised as having an overall ‘uncertain risk’. The overall certainty of the evidence was ‘very low’. The results indicate that topical and systemic propolis may decrease the duration of healing, alleviate pain, and reduce redness in patients with RAS compared to a placebo. However, the certainty of the evidence is very low. These may be due to the high risk of bias, substantial heterogeneity, and limited sample sizes in the included studies. For these reasons, the results of this review should be interpreted with caution. Nevertheless, the limited number of adverse effects observed suggests that propolis may have a favourable safety profile when used for a short period in treating RAS.

## 1. Introduction

Recurrent aphthous stomatitis (RAS) is a multifactorial, debilitating disease with three primary varieties: minor, major, and herpetiform [1,2]. Minor RAS, also called Miculiz’s aphthae, represents the most common form, with lesions ranging from 8 to 10 mm. These typically occur on the non-keratinized lining of the mouth and heal without scarring in 10–14 days [3]. Major RAS (periadenitis mucosa necrotica recurrens or Sutton’s disease) produces lesions larger than one centimetre in diameter, which may persist for up to six weeks and recur for decades, often resulting in scarring after healing. These lesions generally manifest after puberty. Herpetiform RAS is characterised by recurrent crops of small ulcers (2–3 mm) that may coalesce into larger, irregular ulcers with extended persistence. Although these ulcers resemble herpes simplex viral infections, they are not preceded by blisters or vesicles and lack viral particles. Typically, these ulcers resolve without scarring in 10–14 days [4,5].

Bechet’s disease (BD), a systemic vascular disorder, is marked by recurrent oral aphthous ulcers, genital ulcers, erythema nodosum-like nodules, purpuric patches, thrombophlebitis, and inflammation of the central nervous system and eyes, all of which can significantly impact morbidity and mortality [6].

The prevalence of RAS varies globally and between different age groups, with estimates ranging from 0.9 to 78% of the population [7]. Such variations may be attributed to differences in study design, investigative methods, and diagnostic criteria [8]. As early as 1989, Scully and Porter [5] suggested that demographic and environmental factors could influence the prevalence of RAS. Studies have noted an association between RAS frequency and age, suggesting a higher incidence in younger populations [9,10]. Although major RAS is more prevalent among younger individuals, herpetiform RAS typically occurs in adulthood, with the severity of symptoms and frequency of episodes diminishing with age [8].

The aetiology of RAS remains elusive, but Lewkowicz et al. 2008 [11] postulated that oral epithelial ulceration in patients affected by RAS could be due to a T-cell-mediated immune response, as evidenced by a high concentration of T cells in lesions and corroborated by reports of elevated cytokine levels and Th1-related gene overexpression [12,13,14].

In addition to immune and inflammatory factors, trauma, microorganisms, and dietary elements can disrupt immune regulation and precipitate RAS [15]. Genetic predispositions can also weaken mucosal immune responses to oral stimuli, leading to inflammation and the appearance of RAS [16].

Bees produce propolis, a resinous substance used for construction and maintenance within the hive, which possesses anti-inflammatory, antioxidant, antibacterial, antimycotic, antifungal, antiulcer, anticancer, and immunomodulatory properties [17]. Propolis has been used to treat various medical conditions, including those affecting the gastrointestinal tract [18], gynaecological health, neoplastic conditions [19], skin [20], and oral health [21]. The bioactive compounds in propolis promote wound healing [17] and exhibit analgesic effects, primarily attributed to its rich content of bioflavonoids, vitamins, and minerals [22].

There is no definitive cure for RAS, with management strategies focusing on pain relief and improving ulcers. Initial treatment typically involves topical medications, progressing to systemic or laser therapies if necessary [2,23]. Regimens may include steroids, anti-inflammatories, analgesics, antibiotics, antiseptics, immunosuppressants, immunomodulators, and immunopotentiators to alleviate symptoms and reduce the recurrence and severity of the injury. However, these treatments can lead to adverse effects, such as mucosal inflammation.

The preference for traditional, complementary, and alternative medicine (TCAM), such as propolis, over Western medicine is influenced by various factors, including alternative therapies’ perceived safety and effectiveness. According to a recent study [24], the prevalence of TCAM use in 32 countries is approximately 26%. Despite the increase in popularity, there is a paucity of peer-reviewed scientific literature on the efficacy and safety of TCAMs and no guidelines for their use. For these reasons, the findings of clinical trial results should be interpreted with caution and should refrain from being used as the only source for clinical decision-making and recommendations. Instead, clinical decision-making should be based on methodological rigour, including original trial design, data integrity, and potential conflicts of interest. This review aimed to provide a complete evidence profile for propolis concerning the efficacy and safety of treating RAS. The objective was to assess the efficacy and safety of propolis in decreasing pain, healing time, and recurrence rates compared to conventional, alternative, no treatment, or placebo in patients with RAS. The review also aimed to establish the effects of propolis on redness (erythema) and the safety of its use.

## 2. Materials and Methods

This systematic review adhered to the principles outlined in the Preferred Reporting Items for Systematic Reviews and Meta-Analyses (PRISMA) statement and was registered in PROSPERO (registration number: CRD42022327123).

### 2.1. Research Question

The research question for our review was: Is propolis more effective and safer for healing duration, pain, and any preventing adverse effects compared to conventional (corticosteroids) or alternative treatment, placebos, or no treatment in patients with recurrent aphthous stomatitis (RAS)?

### 2.2. Eligibility Criteria

The review team used the population, intervention, comparator/s, and outcomes (PICO) framework to formulate the research question, detailed as follows: Population: Patients of any age or sex with a clinical or histological diagnosis of minor, major, or herpetiform RAS, including cases associated with systemic disorders. Intervention: Any form of treatment containing propolis, whether topical or systemic, such as mouthwashes, ointments, gels, capsules, oral medications, or supplements. Comparison: conventional (usual) treatments, alternative therapies, placebos, or no treatment. Outcome Measures: Primary outcomes included the duration of healing, pain, and adverse effects. Secondary outcomes included the recurrence of ulcers and the degree of redness and exudation.

The review included all randomised controlled trials (RCTs) and quasi-randomised controlled trials (quasi-RCTs) that met the inclusion criteria without restrictions on date, language, or publication status. Exclusions were applied to case reports, case series, observational studies, and studies involving patients with oral ulcers not clinically or histologically diagnosed as RAS.

### 2.3. Study Search Strategy and Process

The review team systematically searched the literature from April to May 2022 in Medline (PubMed), CINAHL (EBSCOhost), Dentistry and Oral Sciences Source (EBSCOhost), Embase (Ovid), the Cochrane Central Register of Controlled Trials (CENTRAL), the WHO International Clinical Trials Registry Platform, clinicaltrials.gov, and ProQuest Dissertations and Theses. To accommodate the inclusion of non-English language studies, the team used electronic translation software. The search strategy, designed explicitly for Medline (PubMed), is illustrated in Supplementary File S1 (Table S1). The research team applied the Cochrane RCT filter to refine the search criteria and adapted the PubMed search strategy for application to the previously mentioned databases (Supplementary File S2). To ensure comprehensive coverage, the team also meticulously reviewed the reference lists of all included studies to identify any additional studies that might not have been detected through electronic database searches.

### 2.4. Study Screening, Selection, and Extraction

The Mendeley referencing management system was used to collect and de-duplicate all potential references. Subsequently, the references were exported to Rayyan [25], where two review authors (T.R. and I.K.) independently conducted further de-duplicated and excluded or included studies. In instances requiring adjudication, another review author (A.S.) provided arbitration. The screening, selection, and data extraction processes unfolded in the following manner: Initially, T.R. and I.K. scrutinised the titles and abstracts against the inclusion criteria. Following this preliminary assessment, the review team obtained and independently evaluated the full texts of potentially eligible studies. The reasons for the study exclusions were systematically documented in a Table of Excluded Studies (Supplementary File S1 Table S2). TR and IK then proceeded to extract data from the included studies using a pre-piloted data extraction form designed in Microsoft Excel., the components are found in Supplementary File S3. Any discrepancies between the two reviewers were resolved through discussion, with AS facilitating consensus when necessary. Furthermore, the authors of the included studies were contacted to retrieve any missing critical data.

### 2.5. Assessment of Risk of Bias

The review incorporated all eligible studies without considering their methodological quality. Two reviewers, T.R. and I.K., independently evaluated the methodological rigour of each included study using the Cochrane “Risk of Bias” tool [26]. A third reviewer, AS, intervened as an arbitrator in divergent assessments to facilitate a consensus. The team categorised the risk of bias for each domain as ‘high’, ‘low’, or ‘unclear’. Two critical domains, allocation concealment and selective reporting, were crucial in determining and ascertaining the overall risk of bias for the studies included in the review.

### 2.6. Assessment of the Certainty of Evidence

The review team evaluated the certainty of the evidence for each outcome using the GRADE approach [27] as ‘high’, ‘moderate’, ‘low’, or ‘very low’ according to five domains: risk of bias, consistency of effect, imprecision, indirectness, and publication bias. The reasons for downgrading the certainty of the evidence for each outcome were documented in the footnotes. The team considered the GRADE ranking to convey findings per outcome and drew on the guidance for informative statements per the Santesso et al. 2020 paper [28].

### 2.7. Measures of Treatment Effect and Data Synthesis

Dichotomous outcomes were recorded as risk ratios (RRs), continuous data as mean differences (MDs), and a 95% confidence interval to accompany all point-effect estimates.

In studies with multiple intervention or control groups, the team selected the pair most relevant to the review question, a treatment and control pair. Data from all time points were included. Missing key outcome statistics from included articles that remained unavailable after contacting the study authors were narratively reported.

Where data permitted, Review Manager 5.4.1 [29] performed a meta-analysis for each outcome, and the results were presented in forest plots. In cases where meta-analysis was not feasible, each outcome’s results were narratively described. Based on a random-effects model, the analysis applied the Mantel-Haenszel method for dichotomous outcomes and the inverse variance method for continuous outcomes.

When a study did not analyse all randomised participants, data from the study’s intention-to-treat (ITT) analysis were prioritised. If a study lacked an ITT analysis, the review team refrained from imputing data and extracted what was available, clearly indicating the absence of ITT data.

The intention was to evaluate the effects of missing data (reporting bias) on the outcomes using funnel plots of outcomes that included five or more studies.

The heterogeneity for each outcome was evaluated by visually inspecting the forest plots for the overlap of 95% confidence intervals and assessing statistical heterogeneity across the included studies using the Chi-square test, I-square statistic, and Tau-square statistic. Heterogeneity was considered substantial if the Chi-square test yielded a *p* value less than 0.1, the Tau-squared statistic was greater than zero, and the I-squared statistic exceeded 50%. Where there was substantial heterogeneity for an outcome and five or more included studies measured that outcome, the team intended to explore the source with subgroup and sensitivity analyses.

The review team planned to explore heterogeneity between studies by conducting subgroup analyses for individuals with RAS, focusing on the type of ulcer (minor, major, or herpetiform), age categories (for example, ≤5 years, 6–17 years, 18–59 years, and 60+ years), and association with Behçet’s disease in outcomes with five included studies.

A sensitivity analysis was planned to evaluate the impact of the risk of bias, using the appropriateness of allocation concealment and the influence of funding on the review findings.

## 3. Results

### 3.1. Characteristics of Included Studies

**Figure 1:**
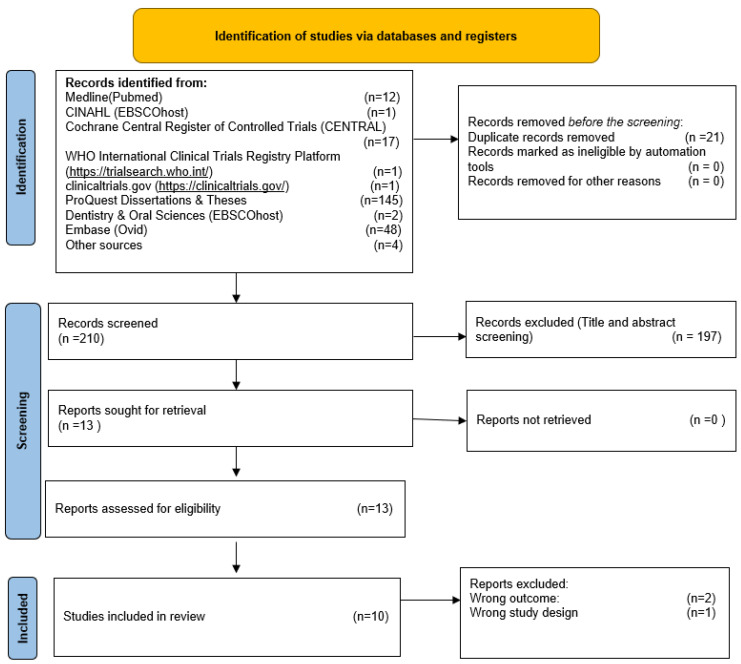
PRISMA flow chart.

This review comprised ten RCTs published between 2003 and 2022 [30,31,32,33,34,35,36,37,38,39]. Figure 1 depicts the PRISMA flow chart.

The included studies comprised 825 participants aged between 18 and 69 years with clinically diagnosed RAS. The duration of the studies in the review varied, ranging from five days to three years, with one study not specifically documenting the duration.

Seven studies evaluated the efficacy and safety of topical propolis [30,31,32,33,34,35,36], and three evaluated systemic propolis [37,38,39]. All except one study [33] was conducted as a single-centre study in a clinical setting affiliated with a university, and only one study failed to explicitly mention where it was conducted [36]. Of the ten studies, one study per location, as indicated, was conducted in China [39], Greece [32], Iraq [30], Morocco [35], Saudi Arabia [33], Sudan [31], and the USA [37], and three studies were conducted in Iran [30,34,36].

One study [36] investigating the efficacy of propolis used propolis as a control, and the remaining studies included propolis as an intervention. All studies, including topical applications, used different propolis formulations in different percentages and carriers. Three studies used propolis as a mouthwash [30,34,36], two as a paste [31,33], and two as an aerosol spray [32,35]. All participants who took systemic propolis (in three studies) took it in tablet form once or twice daily.

Table 1 summarises the included studies and the corresponding authors, and a comprehensive overview is included in Table S3. The summary reflects the total number of participants included in the individual studies. The analyses included participants from the intervention and control groups, which were most appropriate to answer the research question.

### 3.2. Risk of Bias

**Figure 2:**
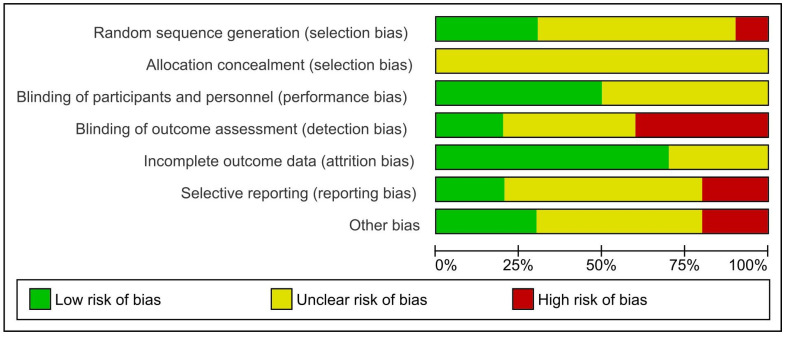
Risk of bias graph: review authors’ judgements about each risk of bias item presented as percentages across all included studies.

We assigned a ‘high’ overall risk of bias rating to two studies [37,39] and ‘unclear’ for the eight remaining studies. (See Figure 2 for the risk of bias graph per domain and Supplementary File S1 Figure S1 for a summary of the risk of bias per study).

One study [36] used systematic sampling and was assigned a judgement of a ‘high’ risk of bias. Three studies [33,34,35] adequately described the randomization process. However, the risk of bias for the remaining six studies remained ‘unclear’ as they did not mention this domain. Allocation concealment was neither described nor sufficient information provided to make a judgement other than ‘unclear’ in all included studies. Six included studies [30,32,33,34,37,38] explicitly reported the blinding of personnel and participants. However, in one study [35], the participants could not be blinded due to the nature of the intervention, and the study was considered a ‘high’ risk. The remaining three studies contained insufficient information to make a judgement other than ‘unclear’. Two studies [33,34] clearly stated that outcome assessors were blinded to the interventions received by participants. However, the authors of four included studies [30,33,38,39] did not provide sufficient information on the blinding of outcome assessors and were judged as ‘unclear’. Studies where it was unclear who measured the outcomes or when outcomes were self-reported by the participants [31,35,36,37], were considered to be at ‘high risk’. A ‘low risk’ of attrition bias was assigned to seven studies [30,33,34,35,37,38,39], and three studies [31,32,36] were judged as having an ‘unclear’ risk of attrition bias due to insufficient information. Three studies [34,35,38] had a “low” risk of reporting bias. Two studies (34,38) reported sufficient information to be classified as ‘low’ risk, while six studies [30,31,32,33,34,36] provided insufficient information on reporting and were judged ‘unclear’. The outcomes of the two studies [37,39] were reported in a manner that compromised the clarity and accuracy of the findings and was assigned a ‘high’ risk.

Three included studies [30,34,38] failed to report essential baseline characteristics per group and were judged as ‘unclear’ risk, while two studies [37,39] did not report the characteristics of individually randomised groups at baseline and were considered ‘high’ risk. The remainder of the studies were.

### 3.3. Quality of Included Studies

The GRADEpro programme was used to assess the level of certainty of our review outcomes.

The summary of findings Table 2 illustrates the effects of topical propolis on RAS in terms of duration of healing and pain relief in days, proportion of patients healed in less than a week, % reduction of ulcer size between one and two days, proportion of participants whose pain resolved between one and two days, % reduction of ulcer size and pain score at six days, reduction in number of lesions (%) at three months, proportion of patients whose pain healed at five days, and erythema levels. The summary of findings in Table 3 summarises information on the effect of systemic propolis compared to a placebo for the outcomes >50% ulcer healing within seven days and >50% relapses (these were the only eligible outcomes measured in the included studies for this comparison).

### 3.4. Effects of Interventions

Comparison 1: Topical propolis compared to placebo or alternative treatment.

Primary outcomes: healing, pain, and adverse events were three key outcomes of interest.

Healing: Healing was grouped into five categories, the first being ‘complete healing in days’. Three studies [33,35,36] reported the days required to heal ulcers in patients receiving propolis. Topical propolis may shorten healing time in days on average, but the evidence is very uncertain (MD −1.92, 95% CI −5.36 to 1.52; n = 154; Analysis, 1.1; Supplementary File S1 Figure S2 indicates a very low certainty of evidence). Considerable heterogeneity was observed between the studies (Chi^2^ = 697.11, degrees of freedom (df) = 2; *p* < 0.00001; I^2^ = 100%).

In the second category, the study authors [30,31] measured the proportion of patients taking propolis whose RAS ulcers healed in less than a week. An analysis of studies suggests that a higher proportion of participants treated with propolis may experience healing of RAS within a week compared to the group receiving a placebo. However, the evidence is very uncertain. (RR 9.64, 95% CI 0.78 to 119.33; n = 99; Analysis 1.2; Supplementary File S1 Figure S3 indicated a very low certainty of the evidence). Significant heterogeneity existed between the studies (Chi^2^ = 4.66, df = 1; *p* = 0.03; I^2^ = 79%).

A third category included the impact of topical propolis on the percentage reduction in ulcer size from day one to day two, and two studies [31,36] showed that there may be a decrease in ulcer size during this period. However, the outcome was very uncertain (RR 5.50, 95% CI 0.02 to 1862.15; n = 119, Analysis 1.3; Supplementary File S1 Figure S4 indicated very low certainty of evidence). The two studies also had substantial heterogeneity (Chi² = 15.86, df = 1, *p* = 0.0001; I² = 94%).

The fourth category comprised only one study [36] reporting on the effects of topical propolis on the percentage reduction in ulcer size in patients with RAS at day six. The evidence suggests that propolis is less likely to cause a reduction in the percentage of ulcer size at six days. However, the evidence from this study is of very low certainty (RR 0.79, CI 0.60 to 1.04; n = 40; analysis 1.4; Supplementary File S1 Figure S5).

The final category included one study [34] that evaluated the effect of propolis on reducing the number of RAS lesions three months after intervention. The results suggest that there may be a higher percentage reduction in the number of RAS lesions three months after an intervention with propolis than with a placebo (RR 5.58, 95% CI 1.88 to 16.51 n = 45, Analysis 1.5; Supplementary File S1 Figure S6, with the certainty of the evidence being very low). The same authors reported that RAS lesions were significantly smaller three months after propolis treatment than the placebo (*p* < 0.001).

Where the data did not allow us to calculate an effect size, the results were narratively reported. One study [32] reported the ‘mean reduction in ulcer size’ after the intervention with propolis and a control group on the third, fifth, and eighth days after treatment. Three days after propolis and the placebo intervention, the mean ulcer size in the propolis group was 3.4 mm (SD: 0.843) versus the control group, which was 4.6 mm (SD: 0.516) at df: 18; t = 3.837 and *p* < 0.001. On the fifth day after intervention, the difference was 1.100 mm (SD: 0.567) vs. 2.200 mm (SD: 0.632) df: 18; t = 4.093; *p* < 0.0006; and on the eighth day after the intervention, the difference was reported as 0 (SD:00) propolis compared to the control group, whose mean reduction in ulcer size was 0.700 mm (SD: 0.674) df: 18; t = 3.279 *p* < 0.004. No 95% CIs were reported in this study.

Pain: Pain was the second key outcome of interest. Seven included studies that reported pain, employed different measurement approaches, and were divided into individual categories.

In the first category, two studies [33,35] measured the days require ed to resolve the pain completely. Our evaluation suggests that while propolis may decrease the average number of days to relieve RAS pain completely, the evidence is very uncertain (MD −4.18, 95% CI −5.59 to −2.77; n = 114; Analysis 1.6; Supplementary File S1 Figure S7 indicated a very low certainty of evidence). Studies included in this category showed high heterogeneity [Chi² = 23.08, df = 1; *p* < 0.00001; I² = 96%].

The second category included two studies [30,31] investigating the proportion of patients who experienced pain resolution between one and two days. Although the results suggest that a higher proportion of participants taking propolis, compared to placebo, experience pain relief between one and two days, the certainty of the evidence is very low (RR 2.91: 95% CI 1.92 to 4.41; n = 99; Analysis 1.7; Supplementary File S1 Figure S8). No heterogeneity was detected between the two studies (Chi² = 0.92, df = 1; *p* = 0.34; I² = 0).

The third category contained a single study [30] that focused on the proportion of participants whose pain resolved on day five. Our analysis failed to detect a difference between the 1% concentration of propolis mouthwash and a mouthwash composed entirely of distilled water to alleviate pain at this timepoint; however, the level of certainty is very low (RR 1.60: 95% CI 0.80 to 3.20; n = 20; Analysis 1.8; Supplementary File S1 Figure S9).

The next category included one study [36] reporting on the change in pain score from baseline to day six. Our findings suggest that, on average, there were no changes in pain score between participants who used propolis compared to placebo on day six (MD 1.0, 95% CI −2.18 to 4.18; n = 20; Analysis 1.10; Supplementary File S1 Figure S10 indicated a very low certainty of evidence).

Two studies did not provide raw data in their manuscripts. One study [32] focused on the mean strength of pain after the third, fifth, and eighth days after intervention with propolis vs. a calcium-based spray. Eight days after the intervention, the mean strength of pain in the propolis group was 0 (SD:00) compared to the calcium-based spray group, which still experienced a mean pain level of 1.500 (SD: 0.527) df: 18; t = 3.00; *p* < 0.007. No 95% CIs were reported in this study. These results concurred with those of another included study [34] that reported participants experiencing less pain after treatment with propolis compared to a control group after three months (*p* < 0.001).

Adverse events: One patient reported early signs of erythematous mucosal change and complained of itching after topical propolis [30]; no other studies reported adverse or side effects.

Secondary outcomes: Only two studies [34,35] reported the frequency of RAS recurrence in patients using topical propolis. One study [34] showed a statistically significant decrease in the frequency of RAS outbreaks in patients treated with the intervention vs. control groups, with the frequency decreasing from less than once every two weeks to more than once every three months (*p* < 0.00). In a second study [35], a cohort of patients was monitored for six months, during which it was observed that recurring episodes of RAS, which typically manifested monthly, were notably absent. Some patients in this study who were treated with propolis and followed up for six months reported a complete absence of recurrent episodes of RAS that they usually experienced monthly.

A study [33] reported changes in erythema (redness) levels. Our analysis showed that the mean erythema (redness) levels after propolis intervention compared to the placebo or alternative treatment were −2.95 [CI −3.21 to −2.690. n = 64; Analysis 1.11; Supplementary File S1 Figure S11]. This can be interpreted as meaning that, on average, propolis may decrease erythema levels; however, the certainty of the evidence remains very low.

Comparison 2: Systematic propolis compared to placebo or alternative treatment.

The comparison included three studies, two in a meta-analysis [37,39], and the remaining study [38] reported narratively due to the lack of available data for meta-analysis.

Primary outcomes: Our study classified the potential therapeutic benefits of propolis into two distinct categories. The study included in the first category [39] showed that propolis may potentially promote the healing process of more than 50% of RAS lesions in seven days; however, the level of certainty at which it achieves this is very low (RR 0.51: 95% CI 0.37 to 0.70; n = 180, Analysis 2.1; Figure S12).

Narrative: The single study [37] in the second category indicated that there were notable decreases in the average number of ulcers (*p* = 0.02), the duration of healing (*p* = 0.004), and the average size of the ulcers (*p* = 0.001) in participants who received systemic propolis compared to those that received placebo within two weeks of the intervention. Between the beginning and end of the study, the authors noted the following changes: the mean number of lesions (*p* = 0.029), the average healing time (*p* = 0.001), and the mean size of ulcers (*p* = 0.000) using the student T-test.

Pain: Narrative: The only study [38] that assessed the effects of systemic propolis on pain documented the cumulative impacts of propolis compared to placebo in the context of pain reduction. The authors reported a higher reduction in pain from the second intervention session (Z = 0.008). The study also showed consistent and significant improvement from the beginning to the end (Z = 0.003).

*Adverse events*: Only one study [37] reported an adverse effect. One participant in the propolis group developed acne. The remaining studies in this comparison failed to report adverse or side effects experienced from ingesting propolis, indicating a possible favourable safety profile in their respective studies.

*The secondary outcomes:* Secondary outcomes could be grouped into two categories. The study in the first category [37] suggests that individuals treated with propolis may have a higher likelihood of experiencing a greater than 50% reduction in RAS relapses compared to those who received a placebo; however, the certainty of the evidence is very low (RR5.40, 95% CI: 0.79 to 36.68; n= 19; Analysis 2.1 Figure S13).

*Narrative:* One study [38] reported that the mean recurrences of RAS ulcers were reduced to 54% in the propolis group at the end of the study (*p* = 0.001).

### 3.5. Subgroup Analysis

There were insufficient data to perform a subgroup analysis; all ulcers in the study were minor RAS, participant ages ranged from 18 to 60 years, and none of the studies included in the review examined RAS associated with Behçet’s disease.

### 3.6. Sensitivity Analysis and Reporting Bias Assessment

The limited number of studies per outcome precluded a sensitivity analysis and the assessment of reporting bias.

## 4. Discussion

The objective of the review was to evaluate the effects of propolis on healing, pain, recurrence rate, and erythema (redness) in RAS. We also investigated possible adverse reactions in participants who used propolis. There were ten eligible studies, eight of which contributed to the meta-analyses. Two comparisons were addressed with a total effective sample size of 825. The overall risk of bias was judged to be ‘high’ for three studies and unclear for the remaining seven.

Based on the pooled data from seven studies comparing topical propolis with placebo or alternative treatment, the review authors conclude that uncertainty remains about the effects of propolis on the healing, pain, redness, and recurrence rates of RAS. Only one study reported mucosal redness and itching with topical propolis, and another reported acne in the systemic group; therefore, propolis appears safe to use topically and systemically.

A similar systematic review [40] addressing the effectiveness and safety of propolis preparation in treating RAS was subsequently identified. This review included 23 studies, one of which was also included in our investigation [38]. Although the study review question was similar to ours, there were notable differences between our review and that of Jinlong and Wenhau, 2022 [40]. These included the following:

- All articles in their review originated from Chinese medical journals, except one from foreign literature, which we excluded from our study during the title and abstract screening phases as it did not meet our specific inclusion criteria.
- The key distinction between the two studies lies in the respective focuses and criteria for participant selection. The Chinese authors evaluated overall treatment outcomes, including recovery rates, improvement, and any side effects. On the other hand, the current review specifically targeted individuals diagnosed with different forms of ulcers, such as major, minor, or herpetiform RAS. Our study also considered patients with RAS related to broader health conditions while deliberately omitting cases of oral ulcers that are unconfirmed as RAS through clinical or histological examination.
- Another distinction between the two SRs was how propolis was administered in the treatment groups. In the Sr from China, participants in the treatment group received propolis either by itself or with standard medical treatments, with the flexibility to tailor the propolis usage to individual patient needs and conditions. On the other hand, our study was more specific, focusing on propolis in various forms as a stand-alone intervention. Moreover, while the comparison group in the Chinese study was limited to receiving standard medical treatments, the comparison group in our study had a wider range of options, including standard or alternative treatments, placebo, or even no treatment at all.
- The current systematic review incorporated a more diverse international perspective, with one study from China and nine from various countries, including the United States, Greece, Morocco, Iran, and Saudi Arabia. Although the diverse geographical representation offered us a broader contextual framework for assessing the therapeutic impact of propolis on RAS, our study was more susceptible to the limitations previously discussed.
- Our review applied GRADE to assess the certainty of the evidence from the studies included. This is important when conducting systematic reviews to improve the reliability of evidence. However, Jinlong and Wenhau (2022) failed to assess the certainty of the evidence. However, the authors used the ROB2 tool to evaluate the quality of the evidence, whereas our study utilised the ROB 1 tool.
- Jinlong and Wenhua, 2022 [40] reported that propolis was significantly effective in treating RAS with a calculated risk ratio (RR) of 1.40, a 95% confidence interval [1.33 to 1.46], and a *p* value less than 0.00001. However, the authors used the total response rate to indicate the efficacy of treatment. Conversely, the current review covers the degree of redness (erythema) and recurrence rates, each evaluated with distinct endpoints. This methodological approach facilitated a more comprehensive and in-depth understanding of the effect of propolis on RAS, allowing us to discern subtle variations in treatment outcomes.
- Furthermore, this analysis was refined by categorising the selected studies based on the topical and systemic modes of propolis administration. In contrast, the authors of the Chinese review conducted a combined analysis, potentially overlooking potential disparities in treatment responses between the two administration methods.

Both reviews independently reported minimal adverse events associated with topical or systemic use of propolis. This finding reaffirms the possibility of a safety profile for propolis in the context of managing aphthous ulcers.

### 4.1. Implications for Practice

Current evidence does not allow conclusive inferences on the effects of topical propolis compared to a placebo or alternative treatment on ulcer healing in patients affected by RAS. Nevertheless, the intervention may promote better ulcer healing over a more extended period. Furthermore, our observations indicate that short-term use of topical propolis may demonstrate greater efficacy in alleviating pain, reducing redness, and decreasing the frequency of relapses compared to using a placebo in patients with RAS. The systemic administration of propolis showed a more favourable impact on healing, pain management, and the frequency of relapses. However, the evidence for the topical and systemic comparisons and all the outcomes evaluated were very uncertain. Therefore, the current state of the evidence remains inconclusive on the general effectiveness of propolis in treating aphthous ulcers, as the existing limitations of the available evidence base preclude definitive conclusions on the generalizability and applicability of these findings.

### 4.2. Implications for Research

To determine propolis’s true benefit for treating RAS, well-designed, adequately powered RCTs where the propolis source, preparation, compositions, dosages, routes of administration, and reporting are standardised are needed. Future research should also focus on RCTs with larger sample sizes to strengthen the evidence base and provide more robust data on the efficacy of propolis in treating RAS. The majority of studies included in the review lasted relatively short periods. More extensive studies, including those monitoring any potential cumulative adverse effects, are necessary to ensure the safety profile of propolis in long-term use.

#### Limitations

The studies included in this review have various internal and external validity limitations relating to study designs, propolis composition, and preparation.

### 4.3. Limitations in Study Design

- There was often missing or incomplete data, yet we received no responses despite our efforts to acquire incomplete or raw data from study authors. The absence of comprehensive reporting hindered us from assessing the methodological quality of the studies and potentially introduced reporting bias. Essential details such as individual sample sizes per group, confidence intervals, and specifics of randomization and blinding procedures were frequently missing. As a result, a narrative synthesis proved to be the most feasible approach to summarising available evidence based on published data and descriptions.
- The sample sizes between the studies included in this review ranged from 19 to 180 participants. Studies with larger sample sizes were likely to produce more precise estimates of treatment effects. Combining data from studies with varied sample sizes in the meta-analysis may have led to wide confidence intervals and reduced precision in the overall effect estimate. This, in turn, posed a challenge when trying to derive definitive conclusions or accurate predictions regarding propolis efficacy in treating RAS. Furthermore, discrepancies in sample sizes could have contributed to heterogeneity and influenced statistical analysis and data pooling within the meta-analysis. Studies with smaller sample sizes had limited statistical power when detecting minor or moderate treatment effects, increasing the likelihood of type II errors—where a genuine effect may have been overlooked. Moreover, the varying sample sizes may not equally represent the target population of adults who receive propolis for RAS treatment. Smaller studies with restricted sample sizes failed to accurately reflect the diversity within the population under investigation. This factor can potentially compromise the generalizability or external validity of the review’s findings, making it challenging to apply the results across a broader population or draw conclusions about specific subsets within that population.

Studies with smaller sample sizes may have been more susceptible to publication bias. Those with positive results who were overrepresented in the published literature could have biassed the systematic review’s findings, potentially overestimating the effect of treatment.

- It was not possible to assess publication bias due to the limited number of studies. Insufficient data points and small sample sizes prevented our attempts to identify funnel plot asymmetry or use statistical tests to assess the potential effect of publication bias on the review’s validity and comprehensiveness. As a result, publication bias may have caused an overestimation of treatment outcomes and an inadequate understanding of the true effect size of propolis in RAS. Small sample sizes, significant heterogeneity, reporting incompleteness, and the inability to assess publication bias are important challenges in conducting a robust systematic review on the effect of propolis on RAS. These limitations require a cautious interpretation of our findings and emphasise the importance of conducting more extensive, meticulously designed studies with standardised protocols and comprehensive reporting to establish a clearer understanding of the effectiveness of propolis in this context.

### 4.4. Limitations in the Composition of Propolis

The studies included in this review were from various geographical regions with different climates, soil compositions, plant diversity, and sources. Propolis from different regions and plant species has different chemical profiles, concentrations, and bioactive compound quality. These may have led to variations in the composition of propolis and possible inconsistencies in the therapeutic properties and efficacy of propolis products in the RCTs included in the review. Further, the processes involved in collecting, extracting, and preparing propolis can vary between geographical locations, resulting in differences in the final product used in individual RCTs. These discrepancies may have affected various aspects of propolis therapies, such as their bioavailability, stability, and overall effectiveness.

### 4.5. Limitations of Propolis Preparation and Administration

The studies included in the review had topical and systemic routes of administration. Topical propolis was prepared and administered as mouthwashes, pastes, and aerosols, while the systemic route was in the form of tablets. The inclusion of different applications in the reviewed studies may have contributed to the heterogeneous results, as follows:

Including different application methods for propolis in the reviewed studies may have affected its delivery and distribution in the oral cavity. Further, the various ingredients, concentrations, dilutions, and propolis application modes may have led to differences in bioactive substances, pharmacological qualities, and therapeutic benefits. Additionally, variations in extraction methods and solvents used to prepare propolis extract may have affected its structure, bioactive components, and effects on RAS. The compliance and adherence of participants to the recommended usage instructions play a crucial role in the effectiveness of propolis treatment for RAS, and the storage conditions and stability of the propolis formulations may have influenced its efficacy.

## 5. Conclusions

The primary objective of this systematic review and meta-analysis was to comprehensively evaluate the effectiveness and safety of propolis as a treatment for RAS. Our comprehensive search, adhering to PRISMA guidelines, included ten RCTs with 825 participants. The studies varied in their approach, with seven focusing on topical propolis and three on systemic use.

### 5.1. Main Findings

Our findings indicate that in adults with RAS, topical and systemic propolis can potentially reduce the healing duration, pain, and erythema compared to a placebo. However, the evidence currently has very low certainty due to the high risk of bias, significant heterogeneity, and small sample sizes in some studies. Only minimal adverse effects were reported, suggesting a favourable safety profile for propolis use in the short term.

### 5.2. Significance and Implications

The potential of propolis as a treatment for RAS may have some benefits, given its minimal side effects and the limitations of current therapeutic options. However, the variability in study designs, propolis preparations, and measured outcomes limits the generalizability of our conclusions. Clinicians and patients should consider the very low certainty of the evidence when making treatment decisions.

### 5.3. Future Research

There is a pressing need for well-designed, adequately powered RCTs with standardised protocols for propolis source, preparation, composition, dosages, and administration methods. Future studies should also aim for extended follow-up periods to assess propolis’s long-term efficacy and safety. Additionally, research should focus on the impact of propolis on the quality of life of individuals with RAS and its cost-effectiveness as a treatment option.

### 5.4. Limitations

This review acknowledges several limitations, including the heterogeneity of the included studies and the high or unclear risk of bias in many of them. The small number of studies and participants, along with the diverse propolis formulations, also limit the strength of our conclusions.

In summary, while propolis shows potential as a treatment for RAS, the current evidence is insufficient to make definitive recommendations. Further rigorous research is required to establish propolis’s role in managing RAS and to provide patients with safe and effective therapeutic options.

## Figures and Tables

**Table 1:** Summary of included studies.

Topical Propolis											
Author(s) Year (Country)	N	Age (years)	Intervention	Comparator	Type	Study Duration	Dosage and Frequency of Application	Outcome Measures	Time-Points	Unit of Analysis	Overall ROB *
Alemrajabi, et al., 2022 [36] (Iran)	N = 40	18–56 (range)	Pesica	Propolis (30%)	Mouthwash	10 days	15 drops in water thrice daily	Pain intensity, changes in ulcer size	Days 2, 6	Mean	* Unclear
Ali & Rasool, 2011 [31] (Sudan)	N = 120	39.5 (mean)	Propolis in olive oil	Similar formulation without propolis	Paste	8 months	twice daily	Duration of complete ulcer healing, duration of pain disappearance, onset of size reduction	Daily	Percent	* Unclear
Al-Sultan, 2003 [30] (Iraq)	N = 40	29.2 ± 5 (mean)	Propolis (1%)	Distilled water	Mouthwash	5 days	5 mL; thrice daily	Frequency of attacks, Grade of pain, reduction in lesion size = healing	Days 2, 5	Percent	* Unclear
El-Haddad, et al., 2014 [33] (Saudi Arabia)	N = 94	20 to 29. (range)	Commercial honey	Adhesive paste (Orobase ^(R)^)	Paste	8 days	thrice daily	Ulcer size, pain relief, erythema levels	Daily	Mean	* Unclear
Rodriguez-Archilla and Raissoni, 2017 [35] (Morocco)	N = 125	33 ± 12 (mean)	propolis (18%)	Flavoured distilled water	Aerosol	3 years	Spray thrice daily	Disappearance of lesion, the disappearance of pain, Adverse effects	Until the resolution of symptoms	Mean	* Unclear
Stojanovska, et al., 2014 [32] (Greece)	N = 20	20–30 (range)	Proaftol (propolis + essential oils)	Calcium-based supplement	Aerosol	8 days	Spray: 3 to 4 daily	Lesion size, intensity of pain	Days 3, 5, 8	Mean	* Unclear
Tonkaboni, et al., 2016 [34] (Iran)	N = 45	28.18 ± 7 (mean) 18 to 53 (range)	Propolis (3%)	Placebo	Mouthwash	3 months	thrice daily	Pain and burning, size of lesion, frequency of recurrence, healing time	Not explicitly stated	Lesions (%) *p*-Values Z-Values	* Unclear
**Systemic Propolis**											
**Author(s)** **Year** **Country**	**Number of Participants**	**Age** **(years)**	**Intervention**	**Comparator**	**Type**	**Study Duration**	**Dosage and Frequency of Application**	**Outcome** **Measures**	**Time-Points**	**Unit of Analysis**	Overall ROB *
Delavarian et al., 2015 [38] (Iran)	N = 22	28.36 ± 5.75 (mean)	Propolis, sucrose, lactose, and binder in a ratio of 1:6	The same ingredients except for propolis	Tablet	6 months	500 mg once daily	Time of healing Monthly frequency of RAS Size of ulcers Pain level	Every two weeks	Relapses: mean Remainder: means: *p*-Values and Z-Values	* Unclear
Liu and Zhang, 2015 [39] (China)	N = 180	32 (mean) 20 to 45 (range)	Pujia and Propolis	Vitamins	Tablet	10 days	Intervention: twice daily	>50% ulcer healing within 7 days	Days: 3, 7, 10	Percentage	* High
Samet, 2007 [37] (USA)	N = 19	None stated	Propolis	Calcium-based food supplement	Tablet	13 months	500 mg once daily	Frequency of outbreaks Number and severity of outbreaks	Every two weeks	Proportion	* High

* ROB = risk of bias.

**Table 2:** Summary of Findings: Comparison 1.

**Comparison 1: Topical propolis compared to placebo or alternative treatment for treating RAS**						
**Patient or population**: Adults with RAS **Intervention**: Topical propolis **Comparison**: Placebo or alternative treatment						
**Outcomes**	Anticipated Absolute Effects * (95% CI)		**Relative Effect** **(95% CI)**	**№ of Participants** **(Studies)**	**Certainty of the Evidence** **(GRADE)**	**Comments**
	**Risk with** **Placebo or alternative Treatment**	**Risk with Topical Propolis**				
Complete healing in days.	The mean complete healing in days ranged from 5.2 to 8.96	MD 1.92 lower (5.36 lower to 1.52 higher)	-	154 (3 RCTs)	⨁◯◯◯ Very low ^a,b,c^	Topical propolis may, on average, shorten the healing time in days, but the evidence is very uncertain.
Proportion patients healed in less than a week	6 per 100	58 per 100 (5 to 100)	RR 9.64 (0.78 to 119.33)	99 (2 RCTs)	⨁◯◯◯ Very low ^c,d,e^	The evidence is very uncertain about the effect of topical propolis on the proportion of patients healed in less than a week
% Reduction in ulcer size between 1 and 2 days	12 per 100	62 per 100 (0 to 100)	RR 5.50 (0.02 to 1862.15)	119 (2 RCTs)	⨁◯◯◯ Very low ^a,f,g^	The evidence is very uncertain about the effect of topical propolis on the percentage reduction in ulcer size between 1 and 2 days
% Reduction in ulcer size at day 6	95 per 100	75 per 100 (57 to 99)	RR 0.79 (0.60 to 1.04)	40 (2 RCTs)	⨁◯◯◯ Very low ^a,c^	The evidence is very uncertain about the effect of topical propolis on the % reduction in ulcer size at day 6
(%) Reduction in the number of lesions at 3 months	13 per 100	73 per 100 (25 to 100)	RR 5.58 (1.88 to 16.51)	45 (1 RCT)	⨁◯◯◯ Very low ^c,h^	The evidence in very uncertain about the effect of topical propolis on the % reduction in the number of lesions at 3 months
Pain relief in days	The mean pain relief in days ranged from 4.64 to 5.96	MD 4.18 lower (5.59 lower to 2.77 lower)	-	114 (2 RCTs)	⨁◯◯◯ Very low ^d,g, i^	Topical propolis may shorten pain relief in days on average, but the evidence is very uncertain.
Proportion of participants whose pain resolved between 1 and 2 days	28 per 100	81 per 100 (54 to 100)	RR 2.91 (1.92 to 4.41)	99 (2 RCTs)	⨁◯◯◯ Very low ^d,g^	The evidence in very uncertain about the effect of topical propolis on the proportion of participants whose pain resolved between 1 and 2 days
pain score on day 6	The mean change in pain score on day 6 was 0	MD 1 higher (2.18 lower to 4.18 higher)	-	40 (1 RCT)	⨁◯◯◯ Very low ^c,h^	The evidence is very uncertain about the effect of topical propolis on the mean pain score at day 6
Erythema levels	The mean erythema level was 5.88	MD 2.95 lower (3.21 lower to 2.69 lower)	-	64 (1 RCT)	⨁◯◯◯ Very low ^h,j^	The evidence is very uncertain about the effect of topical propolis on the mean erythema levels
Proportion of patient whose pain healed at 5 days	50 per 100	80 per 100 (40 to 100)	RR 1.6 (0.8 to 3.2)	20 (1 RCT)	⨁◯◯◯ Very low ^c,h^	The evidence is very uncertain about the effect of topical propolis on the proportion of patients whose pain healed at 5 days

GRADE Working Group grades of evidence: high certainty: we are very confident that the true effect lies close to that of the effect estimate; moderate certainty: we are moderately confident in the effect estimate;: the true effect is likely to be close to the estimate of the effect, but there is a possibility that it is substantially different; low certainty: our confidence in the effect estimate is limited;: the true effect may be substantially different from the estimate of the effect; very low certainty: we have very little confidence in the effect estimate;: the true effect is likely to be substantially different from the estimate of the effect. * The risk in the intervention group (and its 95% confidence interval) is based on the assumed risk in the comparison group and the relative effect of the intervention (and its 95% CI). CI: confidence interval; MD: mean difference; RR: risk ratio. ^a^ All information is from studies with an unclear or high overall risk of bias. ^b^ Substantial heterogeneity (I^2^ = 100%): too few studies to explore heterogeneity with subgroup analysis. ^c^ The 95% CI for the pooled estimate includes a potentially important benefit and a potentially unimportant harm. ^d^ All information is from studies with an unclear overall risk of bias. ^e^ Substantial heterogeneity (I^2^ = 79%): too few studies to explore heterogeneity with subgroup analysis. ^f^ Substantial heterogeneity (I^2^ = 93%): too few studies to explore heterogeneity with subgroup analysis. ^g^ Very wide CI. ^h^ All information is from a study with an unclear overall risk of bias. ^i^ Substantial heterogeneity (I^2^ = 96%): too few studies to explore heterogeneity with subgroup analysis. ^j^ Optimal information size not met.

**Table 3:** Summary of findings: Comparison 2.

**Summary of findings:**						
**Patient or population:** Treating RAS **Setting:** **Intervention:** Systemic propolis compared **Comparison:** Placebo or alternative treatment						
**Outcomes**	Anticipated Absolute Effects * (95% CI)		**Relative Effect** **(95% CI)**	**№ of Participants** **(Studies)**	**Certainty of the Evidence** **(GRADE)**	**Comments**
	**Risk with Placebo or Alternative Treatment**	**Risk with Systemic Propolis Compared**				
>50% ulcer healing within 7 days	18 per 100	9 per 100 (7 to 13)	RR 0.51 (0.37 to 0.70)	180 (1 RCT)	⨁◯◯◯ Very low ^a,b^	The evidence about the effect of systemic propolis on the proportion of patients who experienced >50% healing of ulcers within 7 days is very uncertain.
>50% Relapses	11 per 100	60 per 100 (9 to 100)	RR 5.40 (0.79 to 36.68)	19 (1 RCT)	⨁◯◯◯ Very low ^a,b^	The evidence is very uncertain about the effect of systemic propolis on the proportion of patients who experienced >50% relapses.

GRADE Working Group grades of evidence: high certainty: we are very confident that the true effect lies close to that of the effect estimate; moderate certainty: we are moderately confident in the effect estimate;: the true effect is likely to be close to the estimate of the effect, but there is a possibility that it is substantially different; low certainty: our confidence in the effect estimate is limited;: the true effect may be substantially different from the estimate of the effect; very low certainty: we have very little confidence in the effect estimate;: the true effect is likely to be substantially different from the estimate of the effect. * The risk in the intervention group (and its 95% confidence interval) is based on the assumed risk in the comparison group and the relative effect of the intervention (and its 95% CI). CI: confidence interval; RR: risk ratio. Explanations: ^a^ All information is from a study with a high overall risk of bias. ^b^ Optimal information size not met.

## Data Availability

The data supporting the findings of this review can be found within this article, as the authors used published data sets.

## Supplementary Materials

The following supporting information can be downloaded at: https://www.mdpi.com/article/10.3390/dj12010013/s1. Supplementary File S1: Figure S1: Summary of Risk of Bias; Figure S2. Forest plot of comparison: Topical propolis compared to placebo or alternative treatment, outcome: Complete healing in days; Figure S3: Forest plot of comparison 1: Topical propolis compared to placebo or alternative treatment, outcome: Proportion patients healed in less than a week. Figure S4: Analysis: 1.3: Forest plot of comparison 1: Topical propolis compared to placebo or alternative treatment, outcome:(%) reduction in ulcer size between one and two days; Figure S5. Analysis 1.4: Forest plot of comparison 1: Topical propolis compared to placebo or alternative treatment, outcome: % reduction in ulcer size on day six; Figure S6. Analysis 1.5: Forest plot of comparison 1: Topical propolis compared to placebo or alternative treatment, outcome: Reduction in number of lesions (%) at three months. Figure S7. Analysis 1.6: Forest plot of comparison 1: Topical propolis compared to placebo or alternative treatment, outcome: Complete pain relief in days. Figure S8. Analysis1.7: Forest plot of comparison 1: Topical propolis compared to placebo or alternative treatment, outcome: Proportion of participants whose pain resolved between one and two days; Figure S9. Analysis 8: Forest plot of comparison 1: Topical propolis compared to placebo or alternative treatment, outcome: Proportion of participants whose pain resolved at day five; Figure S10. Analysis 1.10: Forest plot of comparison 1: Topical propolis compared to placebo or alternative treatment, outcome: Change in pain score at day six; Figure S11. Analysis 1.11: Forest plot of comparison 1: Topical propolis compared to placebo or alternative treatment, outcome: Erythema levels; Figure S12. Analysis 2.1: Forest plot of comparison 2: Systemic propolis compared to placebo or alternative treatment, outcome: >50% ulcer healing within seven days; Figure S13. Analysis 2.2: Forest plot of comparison 2: Systemic propolis compared to placebo or alternative treatment, outcome: >50% Relapses; Table S1: PubMed search strategy; Table S2: Table of Excluded studies; Table S3: Risk of Bias. References [30,31,32,33,34,35,36,37,38,39] are cited in the supplementary materials. Supplementary File S2: Search strategies for additional databases; Supplementary File S3: Data extraction components. Supplementary File S4: Differences between protocol and manuscript.
