# Supplementary material for: Efficacy and Safety of Propolis for Treating Recurrent Aphthous Stomatitis (RAS): A Systematic Review and Meta-Analysis
Source: Dent J (Basel). 2024 Jan 6;12(1):13. doi: 10.3390/dj12010013 (PMC10814165; doi:10.3390/dj12010013)
Supplement: Supplementary file 1 [file dentistry-12-00013-s001.zip › Supplementary File S1.pdf]

## Supplementary File S1

## Figures

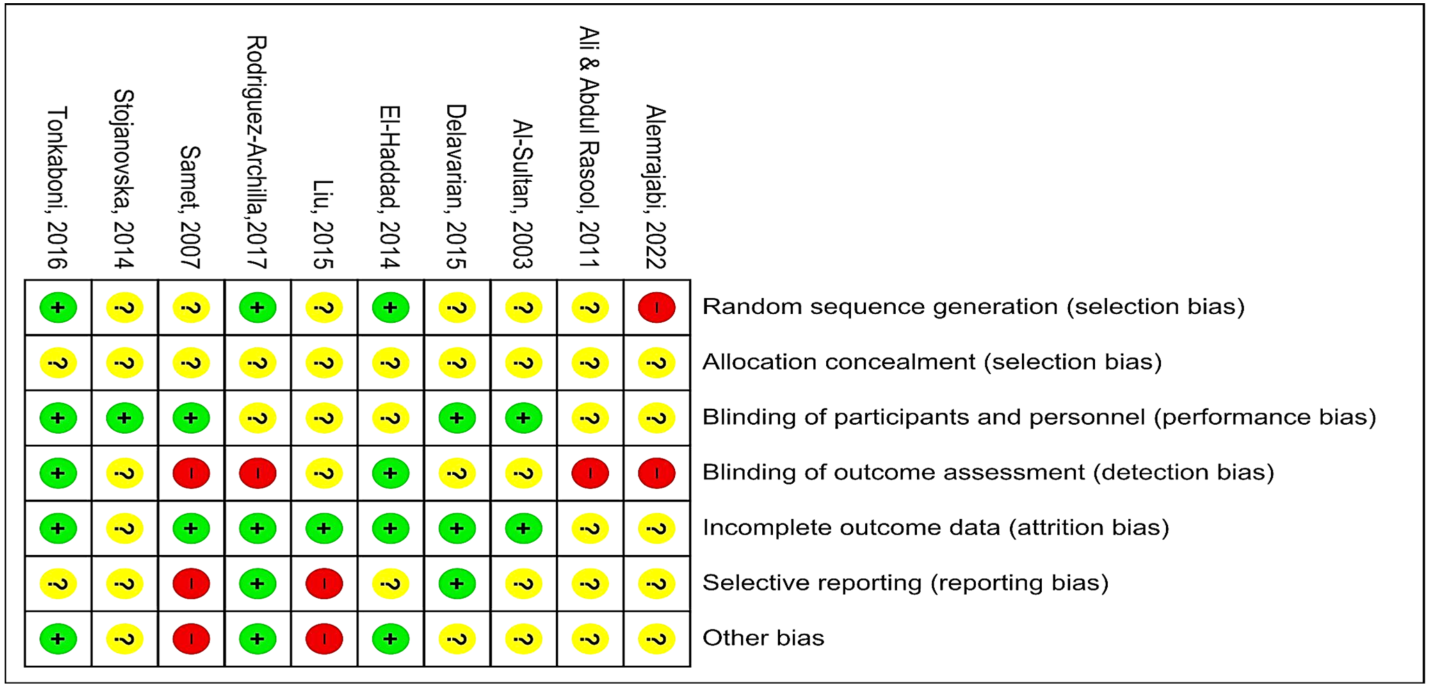


Figure S1: Summary of Risk of Bias

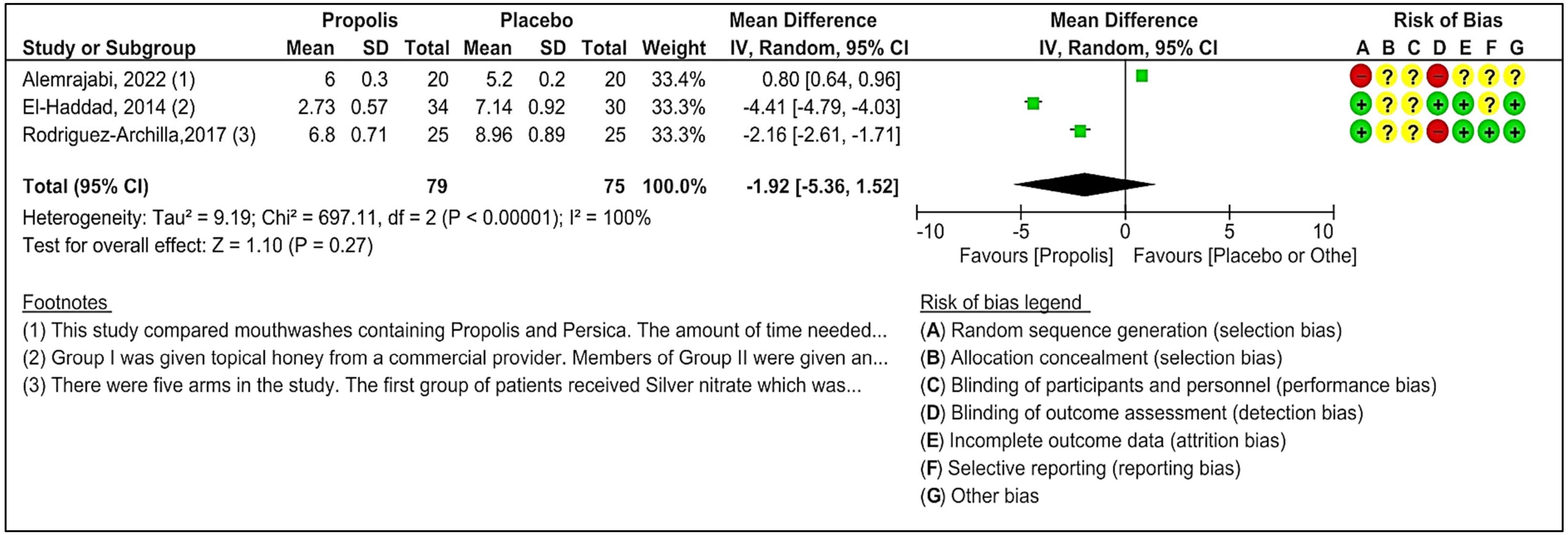


Figure S2: Analysis 1.1. Forest plot of comparison 1: Topical propolis compared to placebo: Complete healing in days

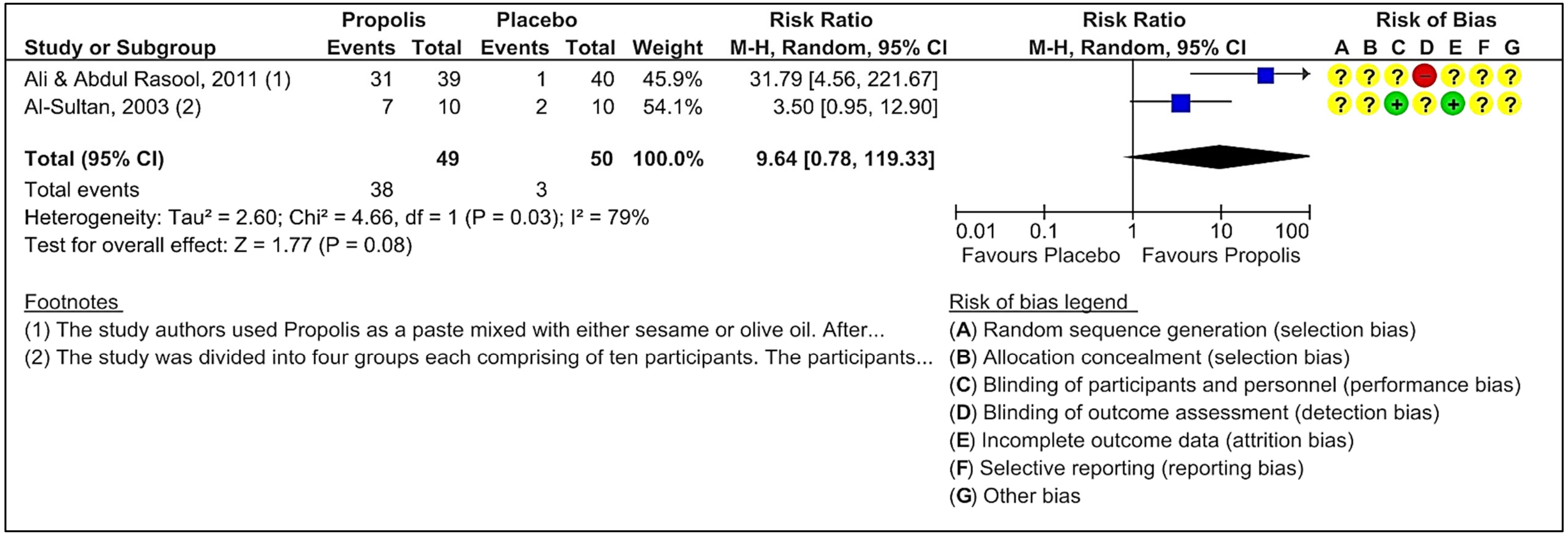


Figure S3: Analysis 1.2: Forest plot of comparison 1: Topical propolis compared to placebo or alternative treatment:  Proportion patients healed in less than a week.

| Study or Subgroup | Propolis Events | Propolis Total | Placebo Events | Placebo Total | Weight | Risk Ratio M-H, Random, 95% CI | Risk of Bias A | B | C | D | E | F | G |
|---|---|---|---|---|---|---|---|---|---|---|---|---|---|
| Alemrajabi, 2022 (1) | 5 | 20 | 7 | 20 | 52.5% | 0.71 [0.27, 1.88] | – | ? | ? | – | ? | ? | ? |
| Ali & Abdul Rasool, 2011 (2) | 25 | 39 | 0 | 40 | 47.5% | 52.27 [3.29, 829.87] | ? | ? | ? | – | ? | ? | ? |
| **Total (95% CI)** | | **59** | | **60** | **100.0%** | **5.50 [0.02, 1862.15]** | | | | | | | |
| Total events | 30 | | 7 | | | | | | | | | | |

Heterogeneity: Tau² = 16.59; Chi² = 15.86, df = 1 (P < 0.0001); I² = 94%
Test for overall effect: Z = 0.57 (P = 0.57)

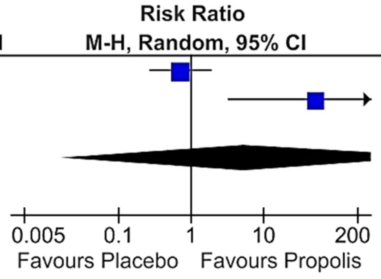


Footnotes
(1) The time points for this study was 2 and 6 days. The current data is the measurement...
(2) The current result is a measure of the proportion of ulcers that reduced during the first day.

Risk of bias legend
(**A**) Random sequence generation (selection bias)
(**B**) Allocation concealment (selection bias)
(**C**) Blinding of participants and personnel (performance bias)
(**D**) Blinding of outcome assessment (detection bias)
(**E**) Incomplete outcome data (attrition bias)
(**F**) Selective reporting (reporting bias)
(**G**) Other bias

Figure S4: Analysis: 1.3: Forest plot of comparison: Topical propolis compared to placebo: (%) reduction ulcer size between one and two days.

| Study or Subgroup | Propolis Events | Propolis Total | Placebo Events | Placebo Total | Weight | Risk Ratio M-H, Random, 95% CI | Risk of Bias A | B | C | D | E | F | G |
|---|---|---|---|---|---|---|---|---|---|---|---|---|---|
| Alemrajabi, 2022 (1) | 15 | 20 | 19 | 20 | 100.0% | 0.79 [0.60, 1.04] | – | ? | ? | – | ? | ? | ? |
| **Total (95% CI)** | | **20** | | **20** | **100.0%** | **0.79 [0.60, 1.04]** | | | | | | | |
| Total events | 15 | | 19 | | | | | | | | | | |

Heterogeneity: Not applicable
Test for overall effect: Z = 1.70 (P = 0.09)

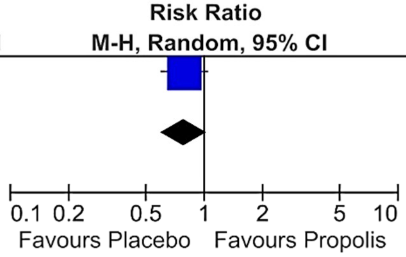


Footnotes
(1) The results refect percentage of reduction size on day 6.

Risk of bias legend
(**A**) Random sequence generation (selection bias)
(**B**) Allocation concealment (selection bias)
(**C**) Blinding of participants and personnel (performance bias)
(**D**) Blinding of outcome assessment (detection bias)
(**E**) Incomplete outcome data (attrition bias)
(**F**) Selective reporting (reporting bias)
(**G**) Other bias

Figure S5. Analysis 1.4: Forest plot of comparison 1: Topical propolis compared to placebo: Reduction in ulcer size on day six.

| Study or Subgroup | Propolis Events | Propolis Total | Placebo Events | Placebo Total | Weight | Risk Ratio M-H, Random, 95% CI | Risk of Bias A | B | C | D | E | F | G |
|---|---|---|---|---|---|---|---|---|---|---|---|---|---|
| Tonkaboni, 2016 (1) | 16 | 22 | 3 | 23 | 100.0% | 5.58 [1.88, 16.51] | + | ? | + | + | + | ? | + |
| **Total (95% CI)** | | **22** | | **23** | **100.0%** | **5.58 [1.88, 16.51]** | | | | | | | |
| Total events | 16 | | 3 | | | | | | | | | | |

Heterogeneity: Not applicable
Test for overall effect: Z = 3.10 (P = 0.002)

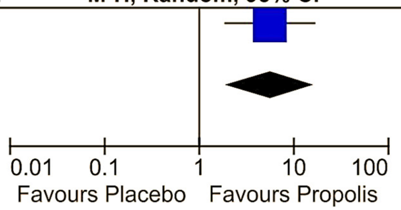


Footnotes
(1) The outcomes were measured at baseline and three months. The intervention...

Risk of bias legend
(**A**) Random sequence generation (selection bias)
(**B**) Allocation concealment (selection bias)
(**C**) Blinding of participants and personnel (performance bias)
(**D**) Blinding of outcome assessment (detection bias)
(**E**) Incomplete outcome data (attrition bias)
(**F**) Selective reporting (reporting bias)
(**G**) Other bias

Figure S6. Analysis 1.5: Forest plot of comparison 1: Topical propolis compared to placebo: Reduction in number of lesions (%) at three months

| Study or Subgroup | Propolis Mean | Propolis SD | Propolis Total | Placebo Mean | Placebo SD | Placebo Total | Weight | Mean Difference IV, Random, 95% CI | Risk of Bias A | B | C | D | E | F | G |
|---|---|---|---|---|---|---|---|---|---|---|---|---|---|---|---|
| El-Haddad, 2014 (1) | 1.04 | 0.21 | 34 | 5.96 | 1.45 | 30 | 48.7% | -4.92 [-5.44, -4.40] | + | ? | ? | + | + | ? | + |
| Rodriguez-Archilla,2017 (2) | 1.16 | 0.37 | 25 | 4.64 | 0.57 | 25 | 51.3% | -3.48 [-3.75, -3.21] | + | ? | ? | - | + | + | + |
| **Total (95% CI)** | | | **59** | | | **55** | **100.0%** | **-4.18 [-5.59, -2.77]** | | | | | | | |

Heterogeneity: $Tau^2 = 0.99$; $Chi^2 = 23.08$, df = 1 (P < 0.00001); $I^2 = 96\%$
Test for overall effect: Z = 5.81 (P < 0.00001)

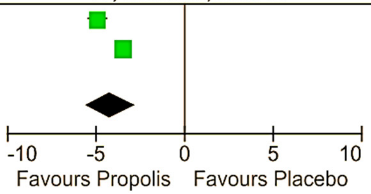


Footnotes
(1) The results in this meta-analysis reflects those reported for the days required for complete pain...
(2) The length of time needed for symptom remission was determined through daily telephone...

Risk of bias legend
(**A**) Random sequence generation (selection bias)
(**B**) Allocation concealment (selection bias)
(**C**) Blinding of participants and personnel (performance bias)
(**D**) Blinding of outcome assessment (detection bias)
(**E**) Incomplete outcome data (attrition bias)
(**F**) Selective reporting (reporting bias)
(**G**) Other bias

Figure S7. Analysis 1.6: Forest plot of comparison 1: Topical propolis compared to placebo or alternative treatment: Complete pain relief in days

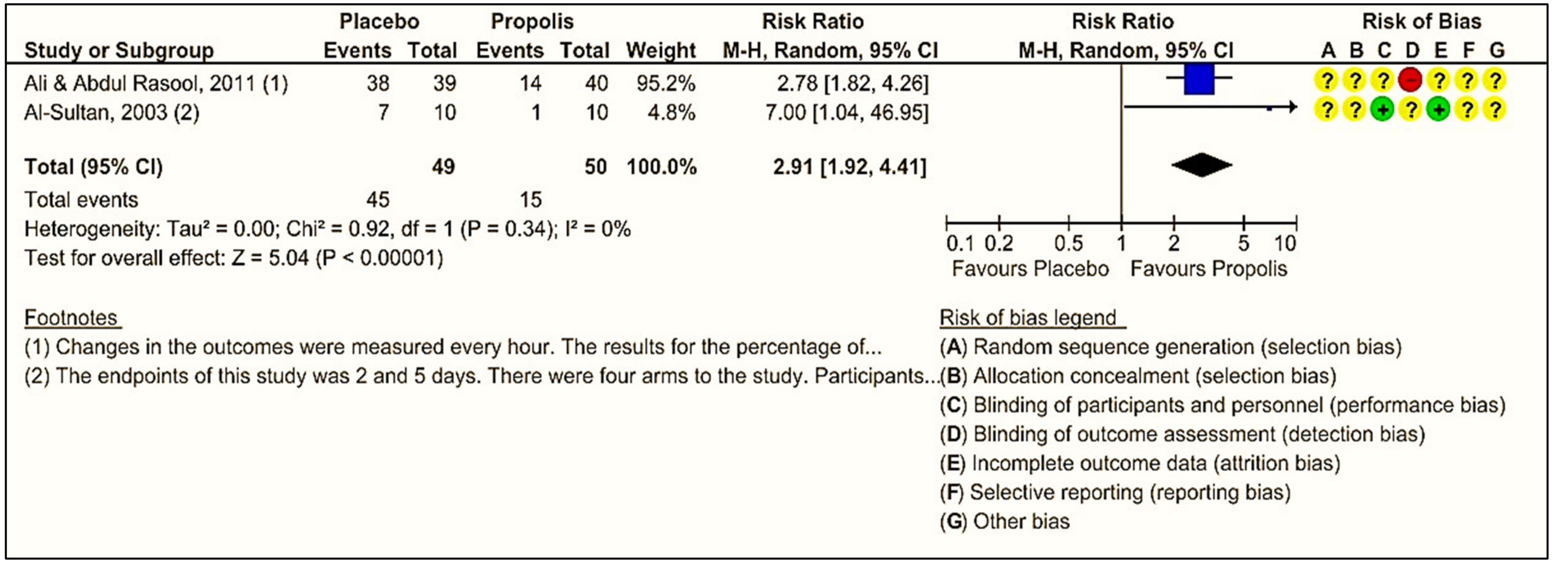


Figure S8. Analysis 1.7: Forest plot of comparison 1: Topical propolis compared to placebo: Proportion of participants whose pain resolved between one and two days

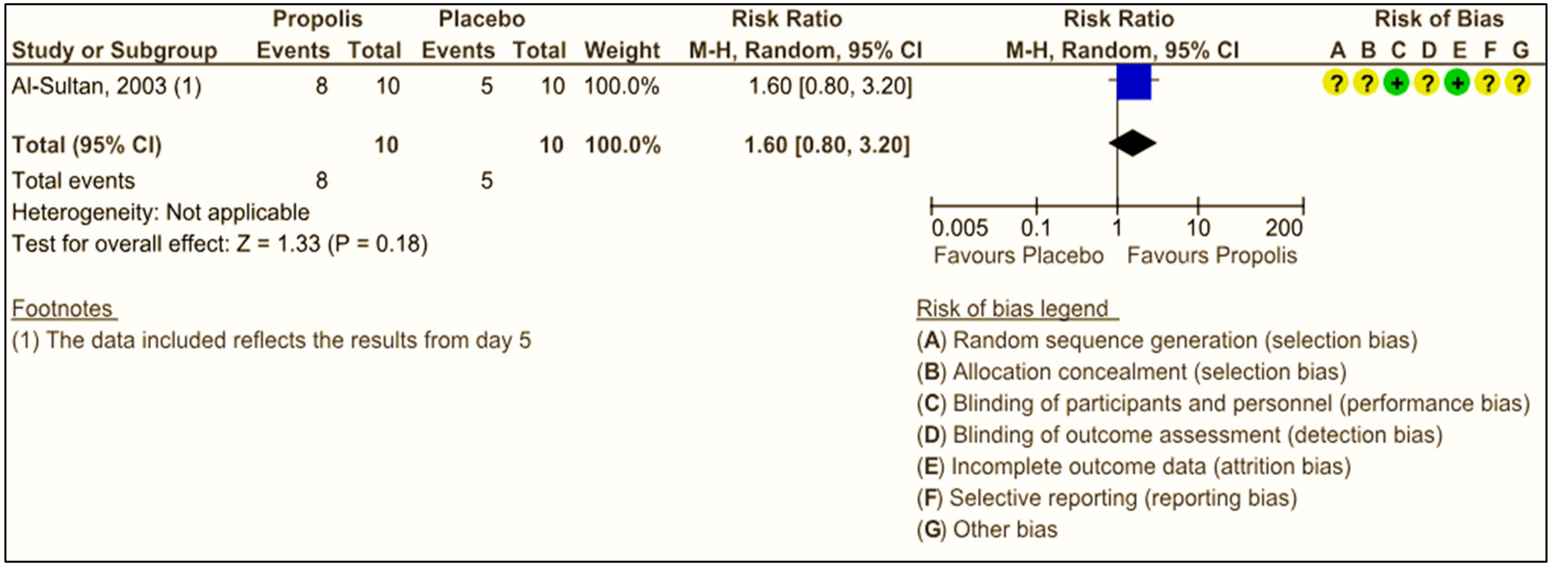


Figure S9. Analysis 1. 8: Forest plot of comparison: 1 Topical propolis compared to placebo: Proportion of participants whose pain resolved on day five.

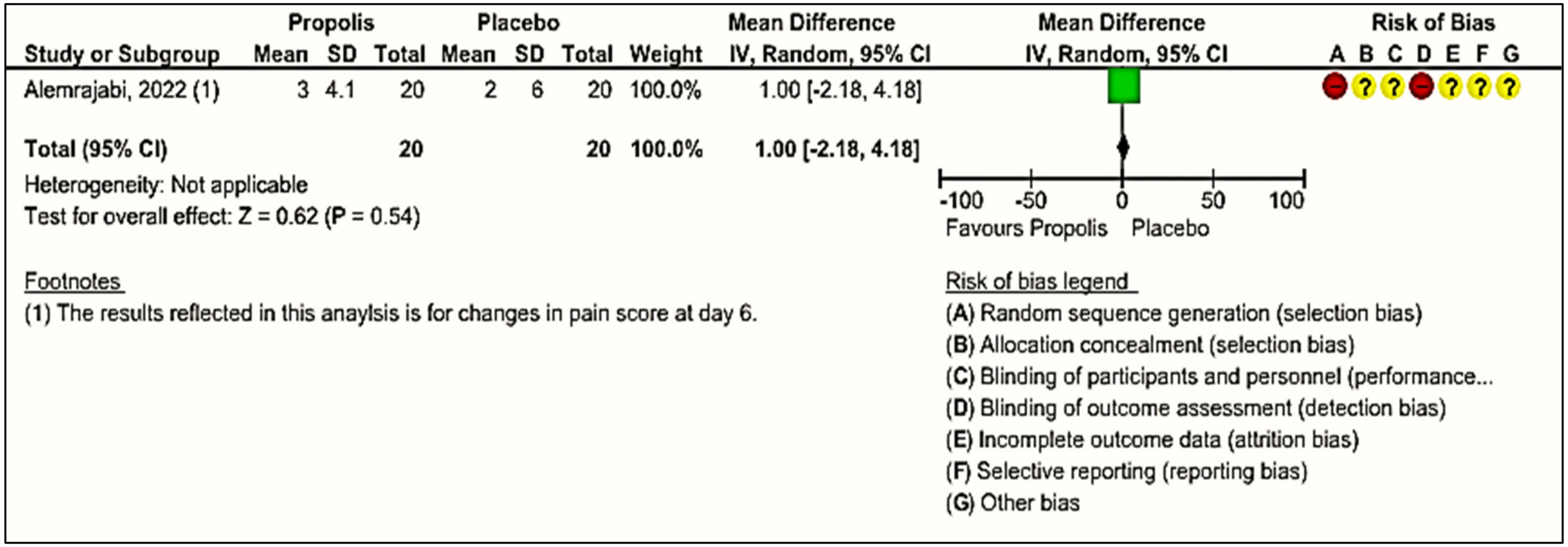


Figure S10. Analysis 1.10: Forest plot of comparison 1: Topical propolis compared to placebo: Change in pain score at day six.

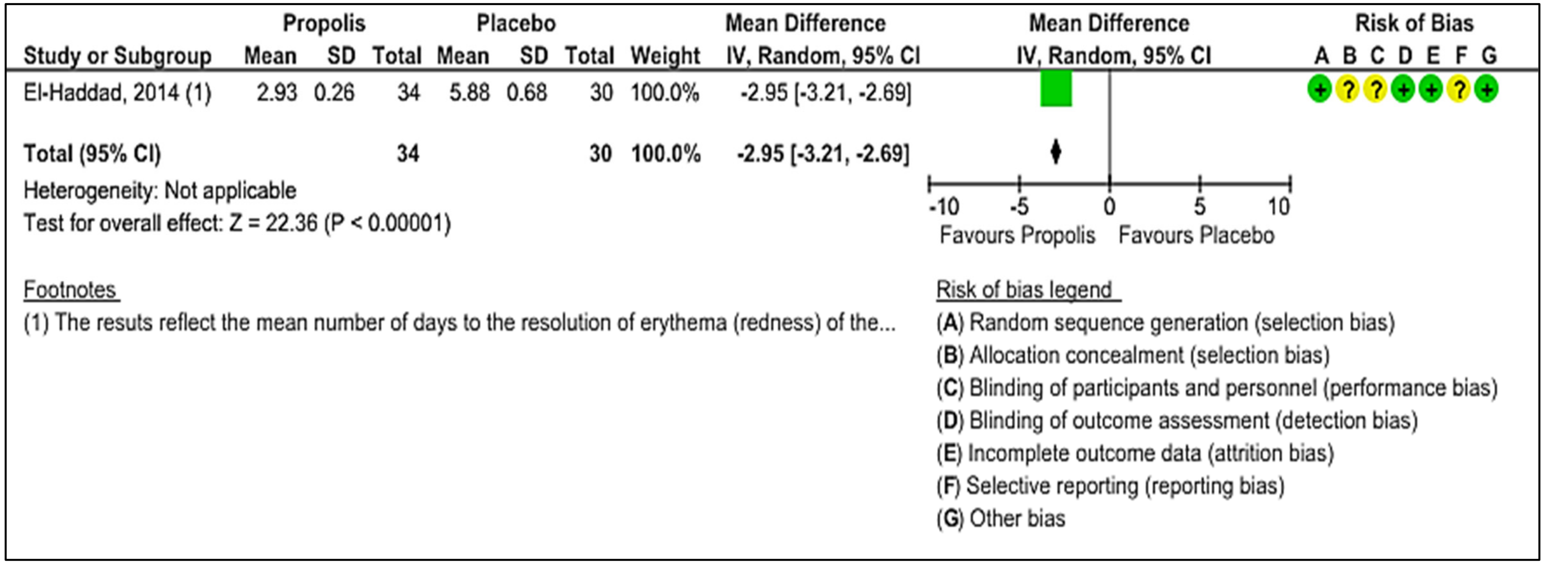


Figure S11. Analysis 1.11: Forest plot of comparison 1: Topical propolis compared to placebo: Erythema levels.

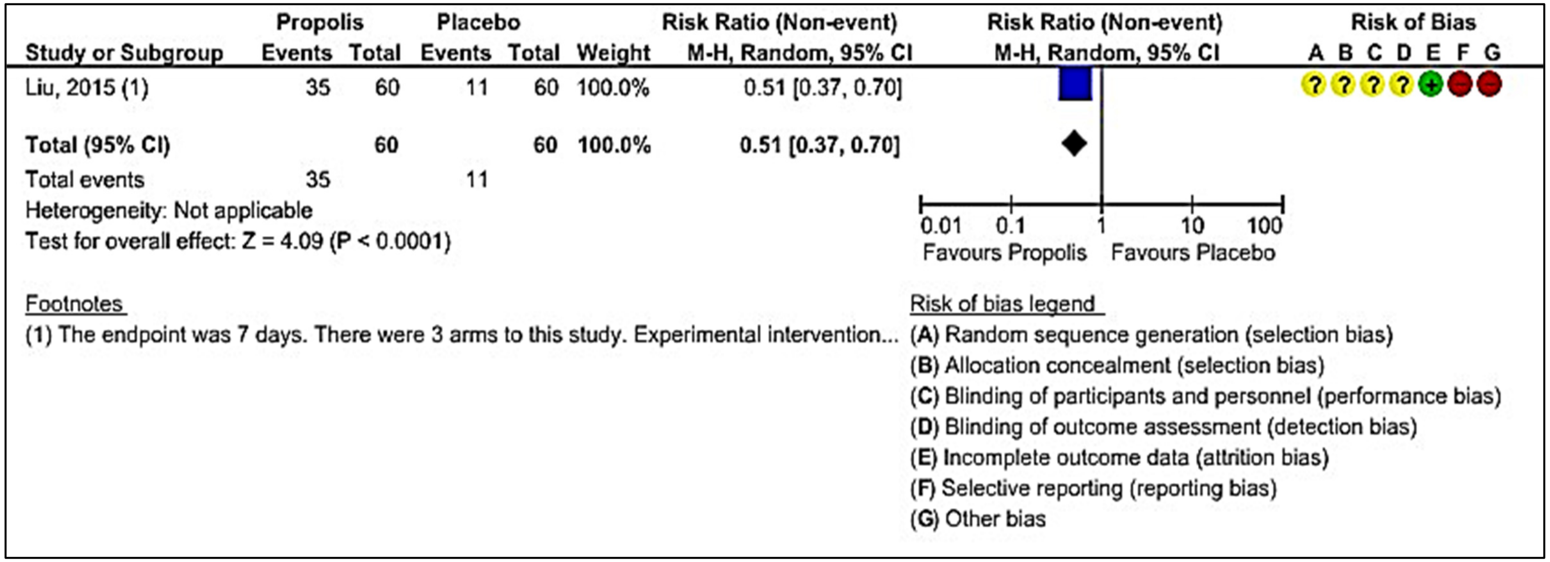


Figure S12. Analysis 2.1: Forest plot of comparison 2: Systemic propolis compared to placebo or alternative treatment, outcome: >50% ulcer healing within 7 days.

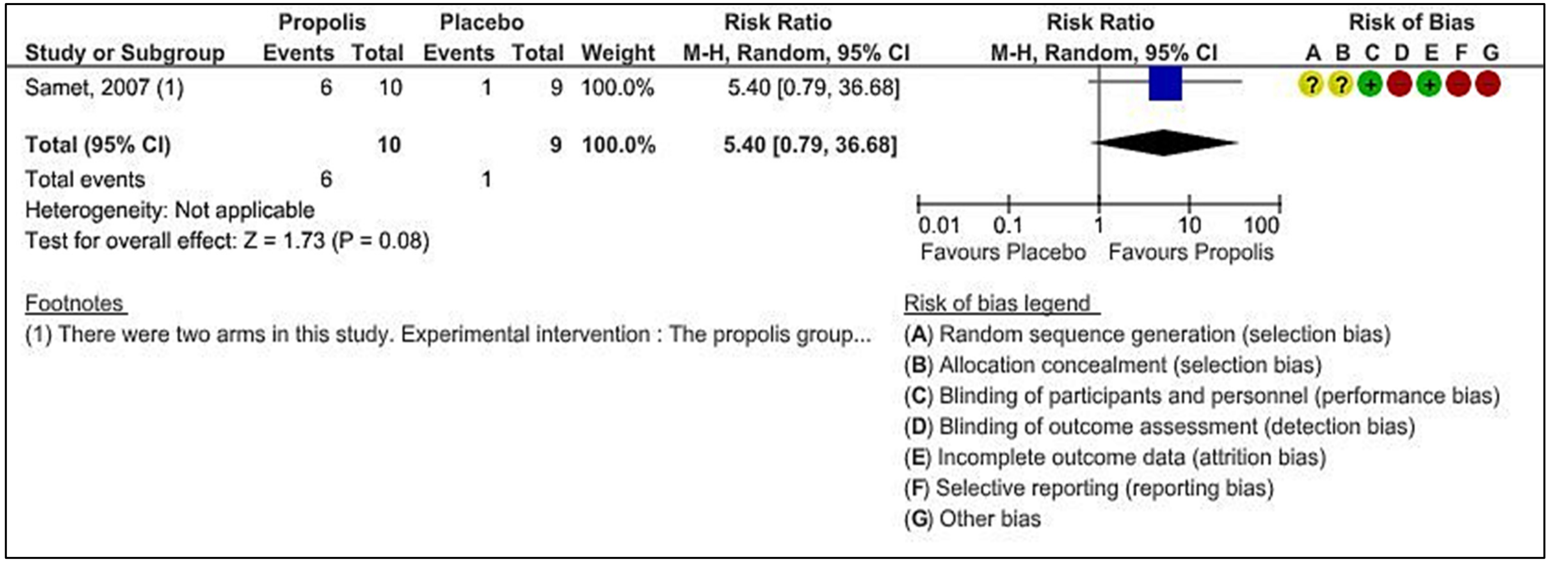


Figure S13. Analysis 2.2: Forest plot of comparison 2: Systemic propolis compared to placebo or alternative treatment, outcome: >50% Relapses

**Supplementary Tables**

**Table S1: Medline (PubMed) search strategy**

Search conducted on 6 May 2022

| Search | Query | Results |
|---|---|---|
| #19 | Search: #18 AND #15 AND #11 | 12 |
| #18 | Search: #16 OR #17 | 4,844 |
| #17 | Search: propolis OR propolin OR "bee glue" OR "bee bread" OR "cera alba" OR galangin OR nivcrisol OR Apitherapy | 4,844 |
| #16 | Search: "Propolis"[Mesh] | 2,504 |
| #15 | Search: #12 OR #13 OR #14 | 45,813 |
| #14 | Search: "canker sores" OR "canker sore" OR "periadenitis mucosa necrotica recurrens" OR "aphthous ulcer" OR "aphthous ulcers" OR "aphthous ulceration" OR "aphthous ulcerations" OR "aphthous stomatitis" OR "aphthous stomatitides" OR "mouth ulcer" OR "mouth ulcers" OR "mouth ulceration" OR "mouth ulcerations" OR "oral ulcers" OR "oral ulcer" OR "oral ulceration" OR "oral ulcerations" OR Sutton's OR Suttons OR Sutton OR Behcets OR Behcet's OR Behcet | 45,813 |
| #13 | Search: "Stomatitis, Aphthous"[Mesh] | 3,535 |
| #12 | Search: "Recurrent aphthous ulceration" OR "Recurrent aphthous ulcer" OR "Recurrent aphthous ulcers" OR "Recurrent aphthous stomatitis" | 1,439 |
| #11 | Search: #9 NOT #10 | 4,720,755 |
| #10 | Search: animals [mh] NOT humans [mh] | 5,002,462 |
| #9 | Search: #1 OR #2 OR #3 OR #4 OR #5 OR #6 OR #7 OR #8 | 5,418,181 |
| #8 | Search: groups [tiab] | 2,378,237 |
| #7 | Search: trial [tiab] | 704,930 |
| #6 | Search: randomly [tiab] | 382,342 |
| #5 | Search: drug therapy [sh] | 2,485,375 |
| #4 | Search: placebo [tiab] | 234,374 |
| #3 | Search: randomized [tiab] | 609,633 |
| #2 | Search: controlled clinical trial [pt] | 658,643 |
| #1 | Search: randomized controlled trial [pt] | 568,682 |

**Table S2: Table of excluded studies**

.

| Author | Reason for exclusion |
|---|---|
| Chiang, M et al., (2021) | Wrong outome |
| Arafa, M et al., (2020) | Wrong outcome |
| Abbasi, A. J et al, (2018) | Wrong study design |

**Table S3: Risk of Bias (ranked according to date)**

**Author: Al-Sultan, 2003 [30]**

| **Bias** | **Authors' judgement** | **Support for judgement** |
|---|---|---|
| Random sequence generation (selection bias) | Unclear risk | Study authors reported that each participant was "assigned randomly" to one of four groups. How the researchers performed the randomisation was not described. |
| Allocation concealment (selection bias) | Unclear risk | The method of concealment was not described. |
| Blinding of participants and personnel (performance bias) | Low risk | "The mouthwash given in pre prepared coded dark bottle …". The instructions for usage were likely the same regardless of group. |
| Blinding of outcome assessment (detection bias) | Unclear risk | It is unclear who measured the various outcomes and whether they were trained with the aim of inter-assessor consistency. |
| Incomplete outcome data (attrition bias) | Low risk | No missing outcome data. There was no loss to follow-up in this study. |
| Selective reporting (reporting bias) | Unclear risk | It is unclear whether there was a study protocol, and no study registration number was reported. All |
| Other bias | Unclear risk | Although the overall age sex, positive family history frequency of RAS attacks, and site of RAS were reported, these were not reported per group to allow comparisons of groups at baseline. |

**Author: Samet, 2007 [37]**

| **Bias** | **Authors' judgement** | **Support for judgement** |
|---|---|---|
| Random sequence generation (selection bias) | Unclear risk | The study authors reported that (quote) "participants were randomly divided into two sub-groups...". However, how the researchers performed the randomisation was not described. |
| Allocation concealment (selection bias) | Unclear risk | The method of concealment was not described. |
| Blinding of participants and personnel (performance bias) | Low risk | Quote:" As this was a double-blinded study, neither the participants nor the investigator. " |
| Blinding of outcome assessment (detection bias) | High risk | The outcomes were (quote): "frequency of outbreaks" and "the duration and subjective severity " were self-reported by the participants. For this reason, it is unclear whether they were trained with the aim of inter-assessor consistency. |
| Incomplete outcome data (attrition bias) | Low risk | Although two patients withdrew from the study and a further two did not complete the study, quote " ...all values were included in the analysis.". |
| Selective reporting (reporting bias) | High risk | The Institutional Review Board approved the study for Human Studies. However, whether the trial was registered with national or international trial registry is unclear. All study outcomes were mentioned in the Methods section. However, not all were reported in the Results section. The frequency of outbreaks was measured as >50% reduction in the frequency of outbreaks. This makes it difficult to compare the raw data of the two groups. Further, the outcome: "the duration and subjective severity" was reported as a quality of life score.' |
| Other bias | High risk | No baseline data was available. |

**Author: Ali & Abdul Rasool, 2011 [31]**

| **Bias** | **Authors' judgement** | **Support for judgement** |
|---|---|---|

| | | |
|---|---|---|
| Random sequence generation (selection bias) | Unclear risk | Study authors reported that they quote:"randomly selected" participants for the trial from an outpatient clinic and that they "randomized" participants into one of three groups. How the researchers performed the randomisation was not described |
| Allocation concealment (selection bias) | Unclear risk | The method of concealment was not described |
| Blinding of participants and personnel (performance bias) | Unclear risk | Quote: "A single blind clinical study was carried out ". However, it is not clear whether the different interventions looked the same. The instructions for usage were likely the same regardless of group. Regarding the primary outcomes: duration of complete ulcer healing and onset of size reduction healing time would have been unaffected by a lack of complete blinding, but for the outcome duration of pain disappearance the risk of performance bias could be high in the absence of complete blinding |
| Blinding of outcome assessment (detection bias) | High risk | The study authors did not mention who measured the various outcomes, and it is unclear whether they were trained with the aim of inter-assessor consistency. It is also unclear whether blinding was adequate. The outcome measurements of the duration of pain disappearance, duration of complete ulcer healing and onset of size reduction were likely influenced by the lack of blinding. |
| Incomplete outcome data (attrition bias) | Unclear risk | It is unclear what the sample size per group was for each outcome's analysis. Dropouts per group were somewhat discrepant, namely Group 1: 5/40, 12.5% (Propolis and sesame oil); Group 2: 1/40, 2.5% (Propolis and olive oil); and Group 3: 0% (Placebo and olive oil). |
| Selective reporting (reporting bias) | Unclear risk | It is unclear whether there was a study protocol, and no study registration number was reported. All expected outcomes were described in the Methods section and reported on in the Results section. |
| Other bias | Unclear risk | Although age and sex distribution per group were reported, no P-values for differences between groups were reported and from the provided data, it appears that gender in Group 2 was very differently distributed compared to the other two groups (28/40 versus 21/40 and 20/40). Also, more elderly were in Group 3 compared to Groups 1 and 2 (6/40 versus 2/40 and 2/40). In addition, condition severity (e.g. mean ulcer size number of ulcers) per group were not reported. |

**Author: El-Haddad, 2014 [33]**

| **Bias** | **Authors' judgement** | **Support for judgement** |
|---|---|---|
| Random sequence generation (selection bias) | Low risk | Quote: "The 94 consecutive subjects .. were assigned randomly (via a computer-generated number list)"' |
| Allocation concealment (selection bias) | Unclear risk | The method of concealment was not described |
| Blinding of participants and personnel (performance bias) | Unclear risk | Although "… subject assessment was measured and recorded by the same researcher, who was blind regarding the type of treatment applied to the subject." (quote), it is unclear whether the patients were aware of the treatment they received. It is also unclear whether the different interventions looked the same or whether the Instructions for usage were the same regardless of group. Regarding the primary outcomes, size reduction of the ulcers and degree of erythema would have been unaffected by a lack of complete blinding, but for the outcome duration of pain score, the risk of performance bias could be high in the absence of complete blinding. |
| Blinding of outcome assessment (detection bias) | Low risk | Quote" Each subject assessment by the same researcherwho was blind regarding the type of treatment.." |

| | | |
|---|---|---|
| Incomplete outcome data (attrition bias) | Low risk | No missing outcome data (quote): Patients who dropped out of the study evaluations were replaced by other patients." |
| Selective reporting (reporting bias) | Unclear risk | A study protocol was registered with an institutional ethics committee, but no study registration number was reported. All expected outcomes were described in the Methods section and reported on in the Results section. |
| Other bias | Low risk | The study authors reported that at baseline, the primary outcomes were the size reduction of the ulcers, degree of erythema and pain score, and the demographic distribution were 'well matched' between the two centres and groups. These were illustrated in tables accompanied by P-values. |

**Author: Stojanovska, 2014 [32]**

| **Bias** | **Authors' judgement** | **Support for judgement** |
|---|---|---|
| Random sequence generation (selection bias) | Unclear risk | Study authors reported that each participant was "divided" "randomly" to one of two groups. How the researchers performed the randomisation was not described. |
| Allocation concealment (selection bias) | Unclear risk | The method of concealment was not described/ |
| Blinding of participants and personnel (performance bias) | Low risk | Quote: "neither participants nor investigators knew the identity of the drugs distributed.". The instructions for usage were the same regardless of group. |
| Blinding of outcome assessment (detection bias) | Unclear risk | It is unclear who measured the various outcomes and whether they were trained with the aim of inter-assessor consistency. |
| Incomplete outcome data (attrition bias) | Unclear risk | It is unclear whether any participants were lost to follow-up or the sample size per group for each outcome's analysis. |
| Selective reporting (reporting bias) | Unclear risk | It is unclear whether there was a study protocol, and no study registration number was reported. All expected outcomes were described in the Methods section and reported on in the Results section. |
| Other bias | Unclear risk | For the main outcomes, "Lesion size in mm" and "intensity of pain", the study authors reported at "day one" there was no significant difference between the groups. A P-value did not accompany this. Also, no baseline characteristics table shows, for example, the distribution of gender between the intervention and comparator groups, nor were the size of the groups mentioned. |

**Author: Delavarian, 2015 [38]**

| **Bias** | **Authors' judgement** | **Support for judgement** |
|---|---|---|
| Random sequence generation (selection bias) | Unclear risk | Study authors reported that "participants were divided by simple random sampling into two groups (intervention and control). How the researchers performed the randomisation was not described. |
| Allocation concealment (selection bias) | Unclear risk | The method of concealment was not described. |
| Blinding of participants and personnel (performance bias) | Low risk | Quote:" In this triple blind clinical trial study The patients, colleagues prescribing the drug, suppliers and statistician were blind to medication…. same shape, color and size". The instructions for usage were likely the same regardless of group. |
| Blinding of outcome assessment (detection bias) | Unclear risk | Quote: " same shape, color and size". However, it is unclear who measured the various outcomes and whether they were trained for inter-assessor consistency. |
| Incomplete outcome data (attrition bias) | Low risk | Quote: "...22 patients included…were attended all the sessions until the end." |

| | | |
|---|---|---|
| Selective reporting (reporting bias) | Low risk | The authors reported that " the project was approved and registered in the IRCT by IRCT2013072214101N1 code". All expected outcomes were described in the Methods section and reported on in the Results section. |
| Other bias | Unclear risk | Although the overall age and sex were reported, these were not reported per group to allow comparisons of groups at baseline. |

**Author: Liu, 2015 [39]**

| **Bias** | **Authors' judgement** | **Support for judgement** |
|---|---|---|
| Random sequence generation (selection bias) | Unclear risk | Study authors reported that "the 180 patients were randomly divided into Chinese medicine treatment group, Western medicine treatment group and control group, 60 patients each." (quote). How the researchers performed the randomisation was not described. |
| Allocation concealment (selection bias) | Unclear risk | The method of concealment was unclear. |
| Blinding of participants and personnel (performance bias) | Unclear risk | Although the instructions for usage were likely the same regardless of group, it is unclear whether the different interventions looked the same. Regarding the primary outcomes, " healing of ulcers would have been unaffected by a lack of complete blinding, but for the outcome " disappearance of pain" the risk of performance bias could be high in the absence of complete blinding. |
| Blinding of outcome assessment (detection bias) | Unclear risk | The study authors did not mention who measured the various outcomes, and it is unclear whether they were trained with the aim of inter-assessor consistency. It is also unclear whether blinding was adequate. The outcome measurements of " healing of ulcers" and "disappearance of pain" were likely influenced by the lack of blinding. |
| Incomplete outcome data (attrition bias) | Low risk | No missing outcome data. |
| Selective reporting (reporting bias) | High risk | It is unclear whether there was a study protocol, and no study registration number was reported. All expected outcomes were described in the Methods section. However, both outcomes, "healing of ulcers and disappearance of pain" were combined as a single outcome in the Results section. |
| Other bias | High risk | The sample size reported in the title does not correspond to the sample described in the study. The baseline demographic data was described and compared. No outcome measures were recorded at baseline for the individual groups. |

**Author: Tonkaboni, 2016 [34]**

| **Bias** | **Authors' judgement** | **Support for judgement** |
|---|---|---|
| Random sequence generation (selection bias) | Low risk | Quote:" Forty-five patients with RAS…..were divided into two groups of intervention (n=22) and control using balanced block randomization method. |
| Allocation concealment (selection bias) | Unclear risk | The method of concealment was not described. |
| Blinding of participants and personnel (performance bias) | Low risk | Quote: "third party was only aware of the codes. The patient, the examiner and … were not aware of the content of bottles (propolis or placebo)." |
| Blinding of outcome assessment (detection bias) | Low risk | Quote: "third party was only aware of the codes. The … the analyzer were not aware of the content of bottles (propolis or placebo)". |
| Incomplete outcome data (attrition bias) | Unclear risk | There are no missing outcome data |

| | | |
|---|---|---|
| Selective reporting (reporting bias) | Unclear risk | Ethics Committee of Tehran University of Medical Sciences and registered in Ir.TUMS.REC1392.654. All expected outcomes were described in the Methods section and reported on in the Results section. Whether the trial was registered in an international or national trials registry is unclear. |
| Other bias | Low risk | The sample size was calculated using statistical methods. Baseline and demographic data was reported on. |

**Author: Rodriguez-Archilla, 2017 [35]**

| **Bias** | **Authors' judgement** | **Support for judgement** |
|---|---|---|
| Random sequence generation (selection bias) | Low risk | Quote: “With the help of a computer program, patients were randomly distributed." |
| Allocation concealment (selection bias) | Unclear risk | The method of concealment was not described. |
| Blinding of participants and personnel (performance bias) | High risk | There was no blinding of patients. Quote: 'The treatments have different administration vehicle; tthe study is not blind the the patients... therefore no blinding of patients. ". However, quote: " The clinician was blind to the treatment received. except in the case of resolution of lesions through silver nitrate." However, it is of the opinion that this incomplete blinding would unlikely affect the outcome " time to remission of lesions" but for the outcome "recurrent aphthous stomatitis symptoms" the risk of performance bias could be affected in the absence of complete blinding. |
| Blinding of outcome assessment (detection bias) | High risk | Quote:" The time time to symptom relief was assessed by the physician through daily telephone follow-up, ….ceased". As the patients assessed the outcomes and they knew the intervention they received, the lack of blinding may have influenced the outcomes " time to remission (in days) of both symptomatology and lesions" |
| Incomplete outcome data (attrition bias) | Low risk | No missing outcome data; Quote: “No patient discontinued treatment." |
| Selective reporting (reporting bias) | Low risk | The study protocol was approved by the Human Research Ethics Committee of the Faculty of Dentistry of the University of Granada (Ref.FOD-UGR-012/2013). All expected outcomes were described in the Methods section and reported on in the Results section. |
| Other bias | Low risk | Baseline characteristics were present with their accompanying p-values. The sample size was calculated via statistical methods. A characteristics table shows the distribution of gender between the experimental and control groups. All expected outcomes were described in the Methods section and reported on in the Results section. Note: Even though the recurrence rate was investigated, the authors did not specify whether this was indeed an outcome under investigation. |

**Author: Alemrajabi, 2022 [36]**

| **Bias** | **Authors' judgement** | **Support for judgement** |
|---|---|---|
| Random sequence generation (selection bias) | High risk | Quote:" who were divided into two groups of 20 via systematic random sampling" |
| Allocation concealment (selection bias) | Unclear risk | The authors did not mention whether there was allocation concealment. |
| Blinding of participants and personnel (performance bias) | Unclear risk | Quote: This study was single-blinded and patients and clinicians were unaware of type of the mouthwashes " However, it is not clear whether the two types of mouthwashes looked the same. Instructions for usage were the same. Regarding the primary outcomes: healing duration and size of lesions would have been unaffected by a lack of complete blinding but for the outcomes pain intensity score and reduction of pain intensity the risk of performance bias could be high in the absence of complete blinding. |

| | | |
|---|---|---|
| Blinding of outcome assessment (detection bias) | High risk | Likely, blinding of outcome assessment was not adequate, and it is unclear who measured the various outcomes and whether they were trained to ensure inter-assessor consistency. The lack of blinding likely influenced the outcome measurements of healing duration and size of lesions. |
| Incomplete outcome data (attrition bias) | Unclear risk | It is unclear whether any participants were lost to follow-up or the sample size per group for each outcome's analysis. |
| Selective reporting (reporting bias) | Unclear risk | It is unclear whether there was a study protocol, and no study registration number was reported. All expected outcomes were described in the Methods section and reported on in the Results section. |
| Other bias | Unclear risk | Although for the main outcomes 'intensity of pain and burning' and 'mean ulcer size' the study authors reported at baseline that there were no significant differences between the groups (accompanied by a P-value), there were no baseline characteristics table or description of the gender and age distribution or condition severity (e.g. mean ulcer size, number of ulcers) between the experimental and control groups. |

**References 30-39 are quoted in the main text**
