# Supplementary material for: Efficacy and Safety of Propolis for Treating Recurrent Aphthous Stomatitis (RAS): A Systematic Review and Meta-Analysis
Source: Dent J (Basel). 2024 Jan 6;12(1):13. doi: 10.3390/dj12010013 (PMC10814165; doi:10.3390/dj12010013)
Supplement: Supplementary file 1 [file dentistry-12-00013-s001.zip › Supplementary File S2.pdf]

**Supplementary File S2**:

**Search strategies for additional databases**

**CINAHL (EBSCOhost)**

Search conducted on 6 May 2022 N=1

We extracted the RCT string and its related information from page 40 of the Technical Supplement to Chapter 4: Searching for and selecting studies of the Cochrane Handbook for Systematic Reviews of Interventions Version 6.3. The formal RCT string for CINHAL has been developed for CINAHL Plus, which we did not have access to and adapted for CINHAL.

(MH randomized controlled trials OR MH double-blind studies OR MH single-blind studies OR MH random assignment OR MH pretest-posttest design OR MH cluster sample OR TI (randomised OR randomized) OR AB (random*) OR TI (trial) OR (MH (sample size) AND AB (assigned OR allocated OR control)) OR MH (placebos) OR PT (randomized controlled trial) OR AB (CONTROL W5 GROUP) OR MH (CROSSOVER DESIGN) OR MH (COMPARATIVE STUDIES) OR AB (CLUSTER W3 RCT)) NOT ((MH ANIMALS+ NOT MH HUMAN) OR (MH (ANIMAL STUDIES) NOT MH (HUMAN)) OR (TI (ANIMAL MODEL) NOT MH (HUMAN))) The CINAHL Plus RCT filter search was validated and published in February 2019 by Glanville et al.[42]. The filter was adapted as an API direct feed by Metaxis in October 2019 and results were screened for inclusion in CENTRAL by Cochrane's RCT Classifier and by Cochrane Crowd.

| | | |
|---|---|---|
| S1 | MH randomized controlled trials OR double-blind studies OR single-blind studies OR random assignment OR pretest-posttest design OR cluster sample | 243,561 |
| S2 | TI (randomised OR randomized ) OR AB random* OR TI trial | 455,216 |
| S3 | MH sample size AND AB ( assigned OR allocated OR control ) | 4,333 |
| S4 | MH placebos OR PT randomized controlled trial | 149,926 |
| S5 | AB CONTROL W5 GROUP | 133,057 |
| S6 | MH CROSSOVER DESIGN OR MH COMPARATIVE STUDIES | 451,505 |
| S7 | AB CLUSTER W3 RCT | 452 |
| S8 | S1 OR S2 OR S3 OR S4 OR S5 OR S6 OR S7 | 951,696 |
| S9 | MH ANIMALS+ NOT MH HUMAN | 90,912 |
| S10 | MH ANIMAL STUDIES NOT MH HUMAN | 121,721 |

| | | |
|---|---|---|
| S11 | TI ANIMAL MODEL NOT MH HUMAN | 3,184 |
| S12 | S9 OR S10 OR S11 | 204,521 |
| S13 | S8 NOT S12 | 906,320 |
| S14 | Recurrent aphthous ulceration OR Recurrent aphthous ulcer OR Recurrent aphthous ulcers OR Recurrent aphthous stomatitis | 745 |
| S15 | MH stomatitis aphthous | 0 |
| S16 | MJ stomatitis aphthous | 613 |
| S17 | canker sores OR canker sore OR periadenitis mucosa necrotica recurrens OR aphthous ulcer OR aphthous ulcers OR aphthous ulceration OR aphthous ulcerations OR aphthous stomatitis OR aphthous stomatitides OR mouth ulcer OR mouth ulcers OR mouth ulceration OR mouth ulcerations OR oral ulcers OR oral ulcer OR oral ulceration OR oral ulcerations OR Sutton's OR Suttons OR Sutton OR Behcets OR Behcet's OR Behcet | 8,122 |
| S18 | S14 OR S16 OR S17 | 8,122 |
| S19 | MH propolis | 168 |
| S20 | propolis OR propolin OR bee glue OR bee bread OR cera alba OR galangin OR nivcrisol OR Apitherapy | 946 |
| S21 | S19 OR S20 | 946 |
| S22 | S13 AND S18 AND S21 | 1 |

**Cochrane Central Register of Controlled Trial (CENTRAL)**

Search conducted 6 May 2022 N=17

| | | |
|---|---|---|
| #1 | "Recurrent aphthous ulceration" OR "Recurrent aphthous ulcer" OR "Recurrent aphthous ulcers" OR "Recurrent aphthous stomatitis" | 399 |
| #2 | MeSH descriptor: [Stomatitis Aphthous] explode all trees | 245 |
| #3 | "canker sores" OR "canker sore" OR "periadenitis mucosa necrotica recurrens" OR "aphthous ulcer" OR "aphthous ulcers" OR "aphthous ulceration" OR "aphthous ulcerations" OR "aphthous stomatitis" OR "aphthous stomatitides" OR "mouth ulcer" OR "mouth ulcers" OR "mouth ulceration" OR "mouth ulcerations" OR "oral ulcers" OR "oral ulcer" OR "oral ulceration" OR "oral ulcerations" OR Sutton's OR Suttons OR Sutton OR Behcets OR Behcet's OR Behcet | 3388 |
| #4 | #1 OR #2 OR #3 | 3404 |
| #5 | MeSH descriptor: [Propolis] explode all trees | 126 |
| #6 | propolis OR propolin OR "bee glue" OR "bee bread" OR "cera alba" OR galangin OR nivcrisol OR Apitherapy | 521 |
| #7 | #5 OR #6 | 521 |
| #8 | #4 AND #7 in Trials | 17 |

**clinicaltrials.gov (https://clinicaltrials.gov/)** N=1

Search conducted on 6 May 2022

propolis OR propolin OR "bee glue" OR "bee bread" OR "cera alba" OR galangin OR nivcrisol

AND

"aphthous ulcers" OR "aphthous stomatitis" OR "canker sores" OR "periadenitis mucosa necrotica recurrens" OR "aphthous stomatitides" OR Behcet

propolis OR propolin OR "bee glue" OR "bee bread" OR "cera alba" OR galangin OR nivcrisol OR Apitherapy | Interventional Studies | "aphthous ulcers" OR "aphthous stomatitis" OR "canker sores"OR "periadenitis mucosa necrotica recurrens" OR "aphthous stomatitides" OR Behcet

Applied Filters: ☑ Interventional

**WHO International Clinical Trials Registry Platform ([https://trialsearch.who.int/](https://trialsearch.who.int/))** N=1

Search conducted on 6 May 2022

"aphthous ulcers" OR "aphthous stomatitis" OR "canker sores" OR "periadenitis mucosa necrotica recurrens" OR "aphthous stomatitides" OR Behcet in the Condition

AND

propolis OR propolin OR "bee glue" OR "bee bread" OR "cera alba" OR galangin OR nivcrisol in the Intervention

Recruitment status is ALL

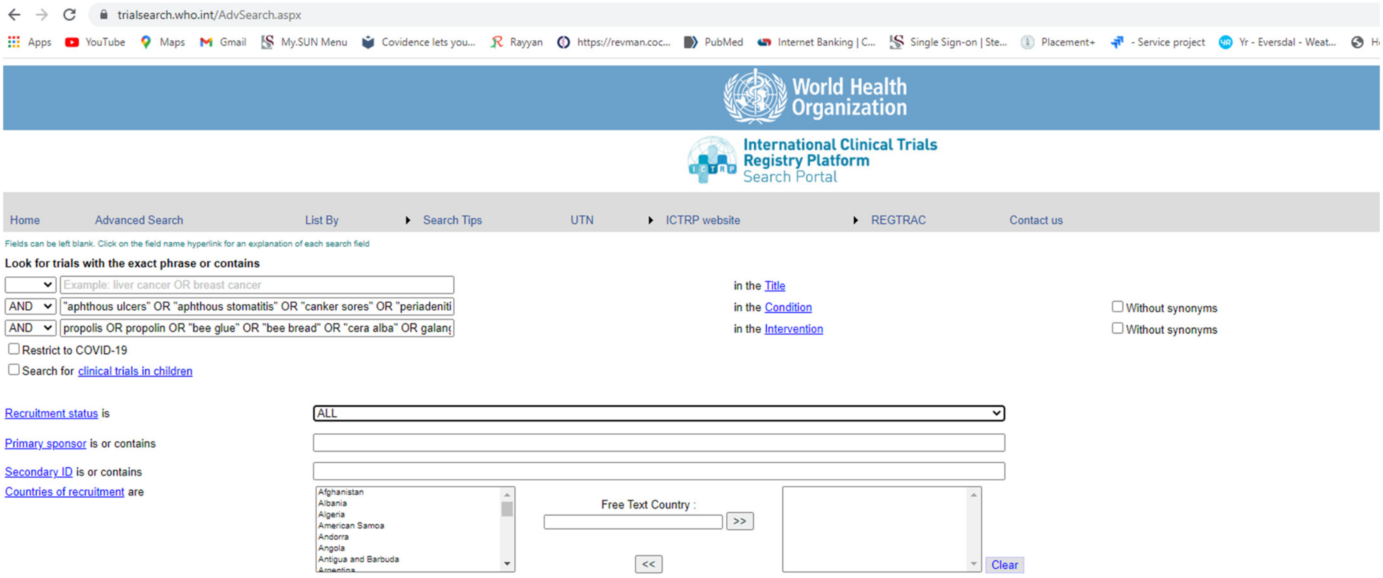


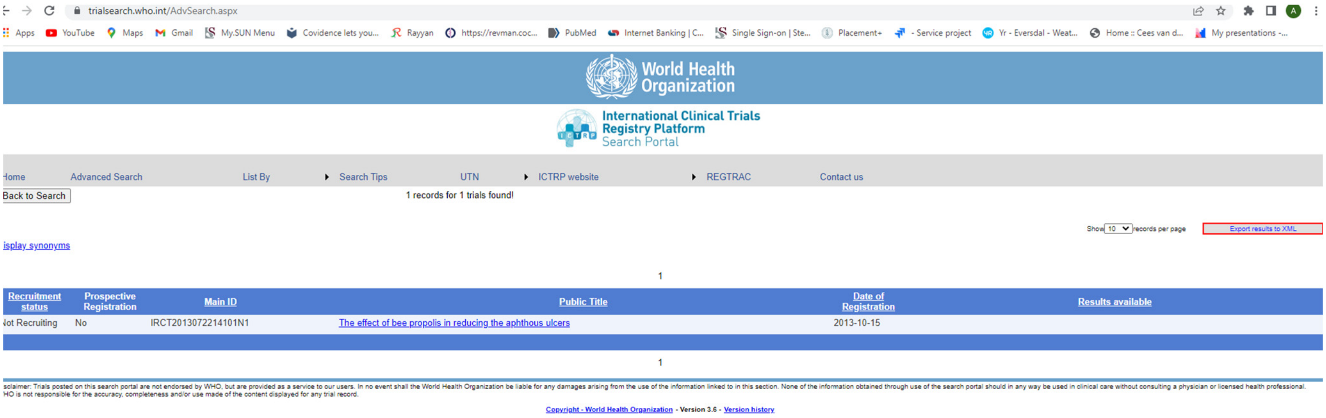

**ProQuest Dissertations & Theses** N=145

Search conducted on 6 May 2022

propolis OR propolin OR "bee glue" OR "bee bread" OR "cera alba" OR galangin OR nivcrisol OR Apitherapy in Anywhere

AND

"canker sores" OR "canker sore" OR "periadenitis mucosa necrotica recurrens" OR "aphthous ulcer" OR "aphthous ulcers" OR "aphthous ulceration" OR "aphthous ulcerations" OR "aphthous stomatitis" OR "aphthous stomatitides" OR "mouth ulcer" OR "mouth ulcers" OR "mouth ulceration" OR "mouth ulcerations" OR "oral ulcers" OR "oral ulcer" OR "oral ulceration" OR "oral ulcerations" OR Sutton's OR Suttons OR Sutton OR Behcets OR Behcet's OR Behcet OR "Recurrent aphthous ulceration" OR "Recurrent aphthous ulcer" OR "Recurrent aphthous ulcers" OR "Recurrent aphthous stomatitis" in Anywhere

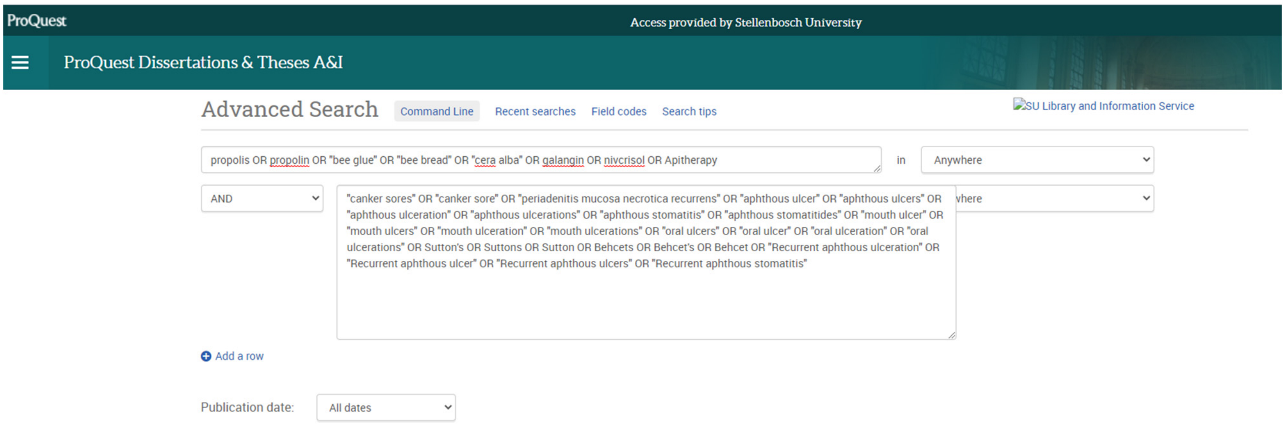

**Dentistry & Oral Sciences (EBSCOhost)** N=2

Search conducted on 08 May 2022

| | | |
|---|---|---|
| S1 | randomized controlled trials OR double-blind studies OR single-blind studies OR random assignment OR pretest-posttest design OR cluster sample | 10,619 |
| S2 | TI ( randomised OR randomized ) OR AB random* OR TI trial | 34,440 |
| S3 | sample size AND AB ( assigned OR allocated OR control ) | 848 |
| S4 | placebos OR PT randomized controlled trial | 3,403 |
| S5 | AB CONTROL W5 GROUP | 20,428 |
| S6 | CROSSOVER DESIGN OR MH COMPARATIVE STUDIES | 7,960 |
| S7 | AB CLUSTER W3 RCT | 19 |
| S8 | S1 OR S2 OR S3 OR S4 OR S5 OR S6 OR S7 | 55,227 |
| S9 | ANIMALS+ NOT MH HUMAN | 0 |
| S10 | ANIMAL STUDIES NOT MH HUMAN | 3,988 |
| S11 | TI ANIMAL MODEL NOT MH HUMAN | 228 |
| S12 | S9 OR S10 OR S11 | 4,136 |
| S13 | S8 NOT S12 | 53,659 |
| S14 | Recurrent aphthous ulceration OR Recurrent aphthous ulcer OR Recurrent aphthous ulcers OR Recurrent aphthous stomatitis | 608 |
| S15 | stomatitis aphthous | 519 |
| S16 | stomatitis aphthous | 519 |
| S17 | canker sores OR canker sore OR periadenitis mucosa necrotica recurrens OR aphthous ulcer OR aphthous ulcers OR aphthous ulceration OR aphthous ulcerations OR aphthous stomatitis OR aphthous stomatitides OR mouth ulcer OR mouth ulcers OR mouth ulceration OR mouth ulcerations OR oral ulcers OR oral ulcer OR oral ulceration OR oral ulcerations OR Sutton's OR Suttons OR Sutton OR Behcets OR Behcet's OR Behcet | 1,736 |
| S18 | S14 OR S16 OR S17 | 1,736 |
| S19 | propolis | 292 |
| S20 | propolis OR propolin OR bee glue OR bee bread OR cera alba OR galangin OR nivcrisol OR Apitherapy | 308 |
| S21 | S19 OR S20 | 308 |
| S22 | S13 AND S18 AND S21 | 2 |

**Embase (Ovid)**

Search conducted on 12 May 2022 N=48

Embase 1947-Present, updated daily

| | | |
|---|---|---|
| 1 | exp propolis/ | 6002 |
| 2 | (propolis or propolin or "bee glue" or "bee bread" or "cera alba" or galangin or nivcrisol or Apitherapy).mp. | 8483 |
| 3 | 1 or 2 | 8483 |
| 4 | exp aphthous stomatitis/ | 5825 |

| | | |
|---|---|---|
| 5 | ("Recurrent aphthous ulceration" or "Recurrent aphthous ulcer" or "Recurrent aphthous ulcers" or "Recurrent aphthous stomatitis").mp. | 1775 |
| 6 | ("canker sores" or "canker sore" or "periadenitis mucosa necrotica recurrens" or "aphthous ulcer" or "aphthous ulcers" or "aphthous ulceration" or "aphthous ulcerations" or "aphthous stomatitis" or "aphthous stomatitides" or "mouth ulcer" or "mouth ulcers" or "mouth ulceration" or "mouth ulcerations" or "oral ulcers" or "oral ulcer" or "oral ulceration" or "oral ulcerations" or Sutton's or Suttons or Sutton or Behcets or Behcet's or Behcet).mp. | 40709 |
| 7 | 4 or 5 or 6 | 40709 |
| 8 | 3 and 7 | 48 |
