# Supplementary material for: Efficacy and Safety of Propolis for Treating Recurrent Aphthous Stomatitis (RAS): A Systematic Review and Meta-Analysis
Source: Dent J (Basel). 2024 Jan 6;12(1):13. doi: 10.3390/dj12010013 (PMC10814165; doi:10.3390/dj12010013)
Supplement: Supplementary file 1 [file dentistry-12-00013-s001.zip › Supplementary File S3.pdf]

Supplementary File S3.
Data extraction components

Identification:

Study details:

- Sponsorship Source
- Country
- Setting
- Comments

Authors contact details:

- Author's name
- Email

Additional identification data:

- Authors' conflict of interest declarations
- Protocol or trial registry entry

Methods:

Unit of randomization:

- Individual
- Cluster

Additional methods data:

- Date of study
- Ethics approval
- Follow-up after the treatment period
- Method of diagnosis
- Sample size calculation

Treatment duration

Population:

Inclusion criteria:

Exclusion criteria:

Group differences:

Additional population data:

Total number of participants randomised

Number of participants randomised per group

Total number of participants completed study

Total number of participants completed experimental group

Total number of participants completed control group

Number of participants lost to follow up in experimental group

Number of participants lost to follow up in control group

Number of participants withdraw from experimental group

Number of participants withdraw from control group

Baseline characteristics:

Characteristics	Experimental intervention	Control intervention	Overall
Gender			
Age			
Type of Ulcer			
Level of Pain experienced			
Duration of Healing			
Frequency of Recurrence			
Adverse effects			
Quality of life			

<u>Interventions:</u>

Characteristics	Experimental intervention	Control intervention
Type of intervention		
Dose		
Route of administration		
Duration		
Frequency		
Concomitant treatment		

<u>Outcomes after intervention:</u>

Characteristics	Experimental intervention	Control intervention	Overall
Type of Ulcer			
Level of Pain experienced			
Duration of Healing			
Frequency of Recurrence			
Adverse effects			
Quality of life			

<u>Outcome measures:</u>

	Experimental intervention	Control intervention

Description		
N		
Mean		
SD		
95% CI		
P-value		
Median		
IQR		
SE		
Narrative		
