# Supplementary material for: Efficacy and Safety of Propolis for Treating Recurrent Aphthous Stomatitis (RAS): A Systematic Review and Meta-Analysis
Source: Dent J (Basel). 2024 Jan 6;12(1):13. doi: 10.3390/dj12010013 (PMC10814165; doi:10.3390/dj12010013)
Supplement: Supplementary file 1 [file dentistry-12-00013-s001.zip › Supplementary File S4.pdf]

Supplementary File S4: Differences between protocol and manuscript

The review protocol for this study was registered in Prospero with the registration number CRD42022327123. During the study, we made some minor adjustments to the original plan. Initially, the inclusion criteria excluded studies involving oral medication, but subsequently, we identified a few additional studies that employed propolis in the form of capsules or oral medication. As a result, we decided to incorporate these studies as a separate comparison in our analysis. In the Methods section, we revised the treatment description under investigation. Initially, the focus was limited to topical applications, such as mouthwashes, ointments, and propolis gels, while injections were excluded. However, we broadened the scope to include capsules and oral medication as eligible treatment forms while excluding injections. In addressing unit of analysis issues, our original intention was to prioritise the longest time point when multiple time points were reported in a study. However, due to the heterogeneity in reporting methods across the studies, we adapted our approach and selected the most appropriate time points to ensure consistency and coherence in the analysis. Efforts were made to handle missing data by contacting the respective study authors for the required information. Unfortunately, we did not receive responses from any of them, and thus, we proceeded with the available data. As there were varying comparator groups, in studies with multiple intervention or control groups, the team selected the pair most relevant to the review question, a treatment and control pair. Regarding the assessment of heterogeneity, we planned to conduct subgroup and sensitivity analyses for outcomes with five or more included studies examining that specific outcome. However, in practice, some outcomes did not have a sufficient number of studies to warrant such analyses. As for evaluating reporting biases, we encountered a limitation as none of the outcomes under consideration were supported by five or more studies, precluding a comprehensive assessment of reporting biases. These modifications were necessary to ensure methodological rigour and address specific issues encountered throughout the review process.
